# In Situ Confinement of 0D Halometallates Within Deep Eutectic Solvents: From Systematic Screening to Metal‐Tunable Luminescence for Anti‐Counterfeiting Eutectogels

**DOI:** 10.1002/advs.76012

**Published:** 2026-06-04

**Authors:** Jeesu Moon, Minkyu Jung, Woojin Jung, Jaeyune Ryu, Jae‐Seung Lee

**Affiliations:** ^1^ Department of Materials Science and Engineering Korea University Seongbuk‐gu Seoul South Korea; ^2^ School of Chemical and Biological Engineering, Institute of Chemical Processes Seoul National University Gwanak‐gu Seoul South Korea

**Keywords:** 0D halometallate, deep eutectic solvent, eutectogel, information encryption, luminescence

## Abstract

Despite the promising microenvrionments of deep eutectic solvents (DESs), including their charge‐rich nature, tunable polarity, and microviscosity, the role of neat DESs as photonic media has remained largely underexplored, often relying on the addition of ex situ‐synthesized luminophores to produce photoluminescence (PL). Here, we report a photostable, metal ion (M^n+^)‐tunable luminescent DES platform driven by the in situ formation of zero‐dimensional (0D) halometallates stabilized within a designed hydrogen bond network. Combinatorial screening of hydrogen bond acceptors and donors identifies an efficient pair for stabilizing highly emissive 0D chloroplumbate(II) complexes under ambient conditions, achieving a PL quantum yield (PLQY) of 60.2%. Moreover, this DES matrix generates versatile emission with customizable excitation and emission wavelengths, PL lifetimes, and PLQYs by accommodating other M^n+^s, including the lanthanides Eu^3+^ and Tb^3+^. Integrating these functional DESs into polymer networks yielded mechanically robust, luminescent eutectogels. By exploiting the independent emissive channels of these soft materials, we successfully constructed a highly multiplexed optical anti‐counterfeiting platform. This systematic work provides insights into the use of DESs as highly dynamic, functional matrices that directly govern the microenvironments for dopant coordination and speciation, leading to the discovery of emergent properties of materials.

## Graphical abstract

We report photostable, photoluminescent deep eutectic solvents (DESs) powered by in situ‐formed zero‐dimensional (0D) halometallates. Stabilized by microenvironment‐controlling hydrogen bond acceptor (HBA) and donor (HBD) networks, these DESs exhibit vibrant, metal ion‐tunable emission (PLQY = 60.2%). Their integration into polymers creates robust eutectogels, whose color combinations unlock a multi‐level optical anti‐counterfeiting platform.

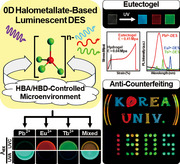

## 1 Introduction

Deep eutectic solvents (DESs) are ion‐rich liquids formed by hydrogen bond donor (HBD) and acceptor (HBA) pairings, featuring depressed melting points and unique physicochemical properties [1, 2, 3]. Their facile preparation, low volatility, and compositional tunability enable diverse applications in separations, catalysis, electrochemistry, and materials synthesis and processing [4, 5, 6, 7, 8, 9, 10, 11, 12]. However, the design space of DESs has been explored predominantly for bulk solvent properties, leaving largely unexplored their potential as functional photonic media that can shape electronic transitions and excited‐state dynamics [13]. In photonics, neat ionic liquids and DESs offer a distinctive opportunity: their charge‐rich nature, tunable polarity, and microviscosity can modulate relaxation pathways, broaden or shift spectra, and enhance photostability by restricting diffusion‐ and collision‐mediated deactivation processes [14, 15, 16, 17, 18, 19]. Despite this promise, systematic studies on how DES composition‐based microenvironments govern photoluminescence (PL) remain scarce [20, 21]. Because canonical HBA/HBD libraries are optimized for liquidity and solvation rather than intrinsic luminophoric function, current PL applications of DESs typically require dissolution of external luminophores or lanthanide/actinide ions into non‐emissive DESs, polymerization‐mediated immobilization, or the use of natural DESs [22, 23, 24, 25, 26, 27, 28, 29, 30]. While these approaches are valuable, they frequently face persistent limitations: (i) external luminophore preparation requires elaborate synthesis; (ii) the DES microenvironments can mask native dye emission; and (iii) conventional organic luminophores remain susceptible to photobleaching and quenching. Consequently, the exploration of DESs as functional photonic media remains significantly hindered. A viable solution is to introduce in situ formed emissive functionality into a systematic DES library [31]. In this approach, excited‐state dynamics are directly regulated by the composition‐dependent microenvironments of the neat DESs, ultimately broadening the limited DES photonics landscape [32, 33, 34].

Zero‐dimensional (0D) chloroplumbate(II) complexes ([PbCl_x_]^2−x^) represent the molecular limit of ns^2^ metal halides in which excitation is localized on an isolated lead halide center and relaxes through strong electron‐phonon coupling into a self‐trapped exciton‐like localized excited‐state [35, 36]. This self‐trapping mechanism is commonly associated with large Stokes shifts, broadband visible PL, and short excited‐state lifetimes (typically ns to sub‐µs) that are attractive for reabsorption‐free light conversion and optical tagging [37]. Extending these favorable traits to the liquid state, however, requires a microenvironment that not only sustains the desired Pb‐Cl coordination but also suppresses competitive solvation and dynamic ligand exchange. In conventional molecular solvents, the distribution of Pb‐Cl complexes can be highly sensitive to halide activity, coordinating donor strength, and trace competitive ligands such as water, which can shift equilibria among PbCl_x_ species and promote ligand exchange, complicating the identification and stabilization of a single emissive chloroplumbate(II) center [38, 39]. This speciation sensitivity can blur structure‐emission correlations in solution, which helps explain why most highly emissive 0D lead halides have been studied as crystalline salts, whereas systematic photophysics of discrete chloroplumbate(II) centers in neat liquids remains comparatively limited [32, 40, 41, 42]. A notable step toward stabilizing liquid‐state 0D emission is the development of luminescent lead halide ionic liquids, where bulky organic cations mitigate crystallization and enable dense populations of emissive chloroplumbate(II) centers [43]. However, these halometallate liquids are typically hygroscopic and require moisture‐controlled handling, underscoring the need for alternative systems that preserve chloroplumbate(II) emission under practical ambient processing. In this context, DESs offer an underexplored opportunity as photonic media because of their heterogeneous polarity and local microviscosity for suppressing nonradiative pathways [16, 44]. Despite the solubility of chloroplumbate(II) in DESs, however, its PL has hardly been explored to date [45]. Herein, we present the construction of an HBA/HBD library to synthesize Pb^2+^‐responsive photoluminescent DESs with bright broadband PL and a high quantum yield (up to 62%), where emissive chloroplumbate(II) centers are generated in situ and dynamically stabilized under ambient conditions. This versatile medium demonstrates multi‐color capacity by hosting lanthanides (Eu^3+^, Tb^3+^) for characteristic red and green emissions. Stoichiometric mixing of these individual channels enables their linear superposition, significantly expanding the tunable color gamut. Crucially, these emission properties are seamlessly translated into the solid state by gelation, yielding mechanically robust and luminescent eutectogels, which are exploited to build multi‐level anti‐counterfeiting platforms for information security.

## 2 Results and Discussion

### 2.1 Library Screening for Pb^2+^‐Responsive Photoluminescent DESs

**FIGURE 1:**
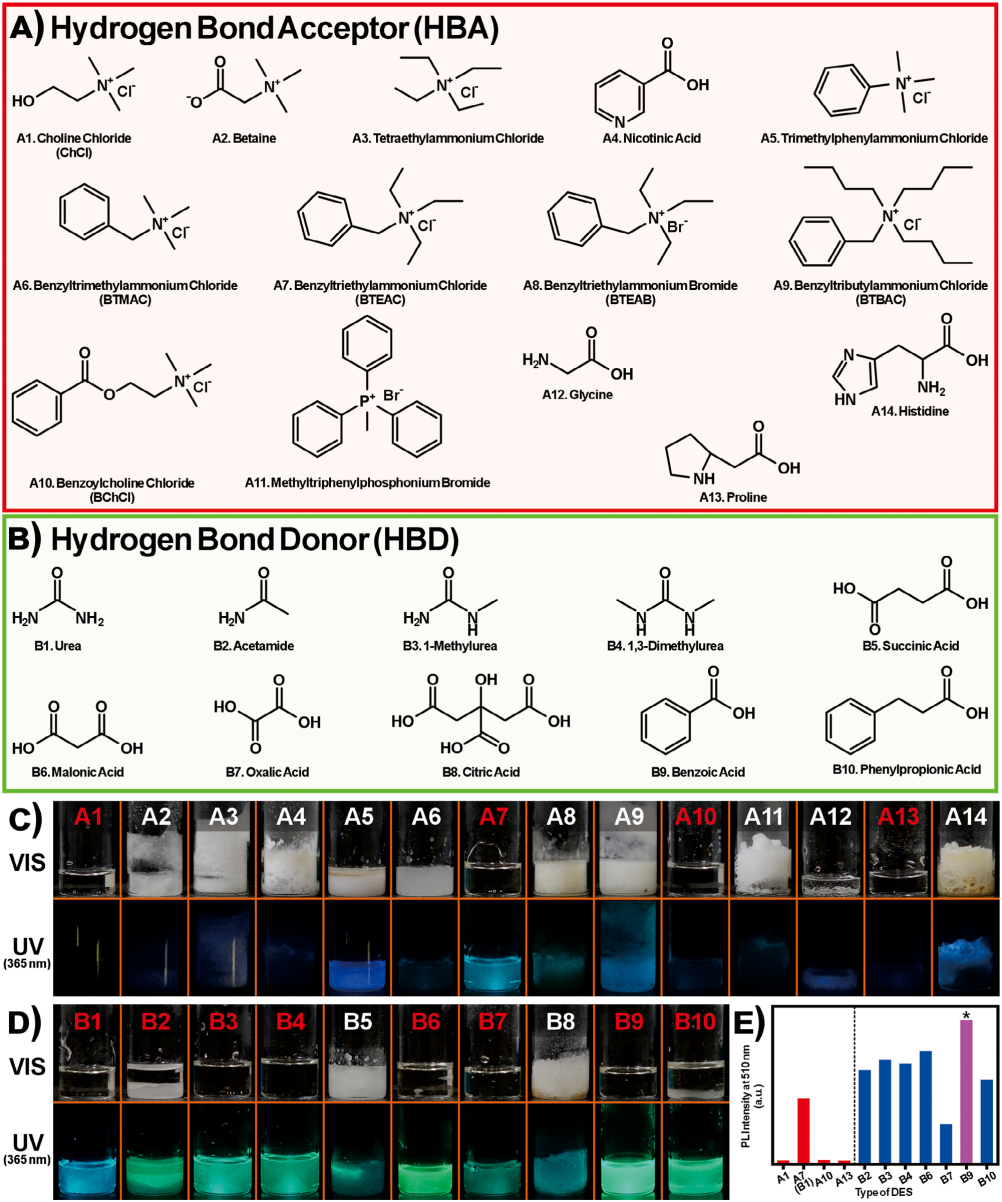
(A and B) Molecular structures of the (A) 14 HBAs and (B) 10 HBDs selected for the investigation of M^n+^‐doped photoluminescent DESs. (C and D) Photographs showing Pb^2+^‐doped combinations of (C) urea (“B1”) with various HBAs and (D) BTEAC (“A7”) with various HBDs after thermal treatment. Top rows: under visible light (VIS); bottom rows: under 365 nm UV irradiation. Red labels highlight combinations which successfully formed homogeneous, single‐phase liquid DESs. (E) Comparison of PL emission intensities at 510 nm for the successfully formed Pb^2+^‐DES candidates under *λ_ex_* = 320 nm. Note that the highest PL intensity is marked with an asterisk.

To identify DESs that become photoluminescent upon metal ion (M^n+^) incorporation, we assembled a library of 14 HBAs and 10 HBDs (Figure 1A,B). The HBAs comprised quaternary ammonium and phosphonium salts (A1‐A3 and A5‐A11), amino acids (A12‐A14), and an N‐heterocyclic compound (A4), whereas the HBDs included urea (B1), urea derivatives (B2‐B4), and carboxylic acids (B5‐B10). Pb^2+^ was selected as the initial probe because its heavy‐atom character and rich coordination chemistry can markedly influence excited‐state relaxation for PL in ion‐rich media [46, 47, 48, 49, 50, 51]. Screening began by pairing urea (B1) with each HBA to evaluate HBA‐dependent DES formation (Figure 1C). Only mixtures containing A1, A7, A10, and A13 formed transparent, homogeneous liquids, while the others remained opaque solids. Among the transparent DESs, only A7/B1, composed of benzyltriethylammonium chloride (BTEAC) and urea, exhibited a measurable Pb^2+^‐triggered PL turn‐on. Subsequently, BTEAC (A7) was paired with the full HBD set under identical conditions (Figure 1D). Most combinations formed nearly homogeneous DESs possessing strong Pb^2+^‐induced PL under UV excitation, except those with B5 and B8. Notably, contrasting behaviors observed in some mixture pairs, such as A7/B1 versus A8/B1, and A7/B5 versus A7/B6, indicated that even a single anion substitution (Cl^−^ vs. Br^−^) or a single methylene group addition can substantially affect liquefaction and DES formation. To compare PL responses quantitatively, we collected PL spectra under identical conditions and extracted emission intensity at 510 nm as a benchmark (Figure 1E and Figure S1). Consistent with visual inspection, the B1‐based DESs derived from A1, A10, and A13 exhibited negligible emission, whereas A7‐based DESs showed Pb^2+^‐responsive pronounced PL turn‐on responses. The BTEAC/benzoic acid (BA; A7/B9) composition produced the highest PL intensity and was therefore selected as the representative photoluminescent DES.

### 2.2 Metal‐Dependent PL and Orthogonal Optical Multiplexing

**FIGURE 2:**
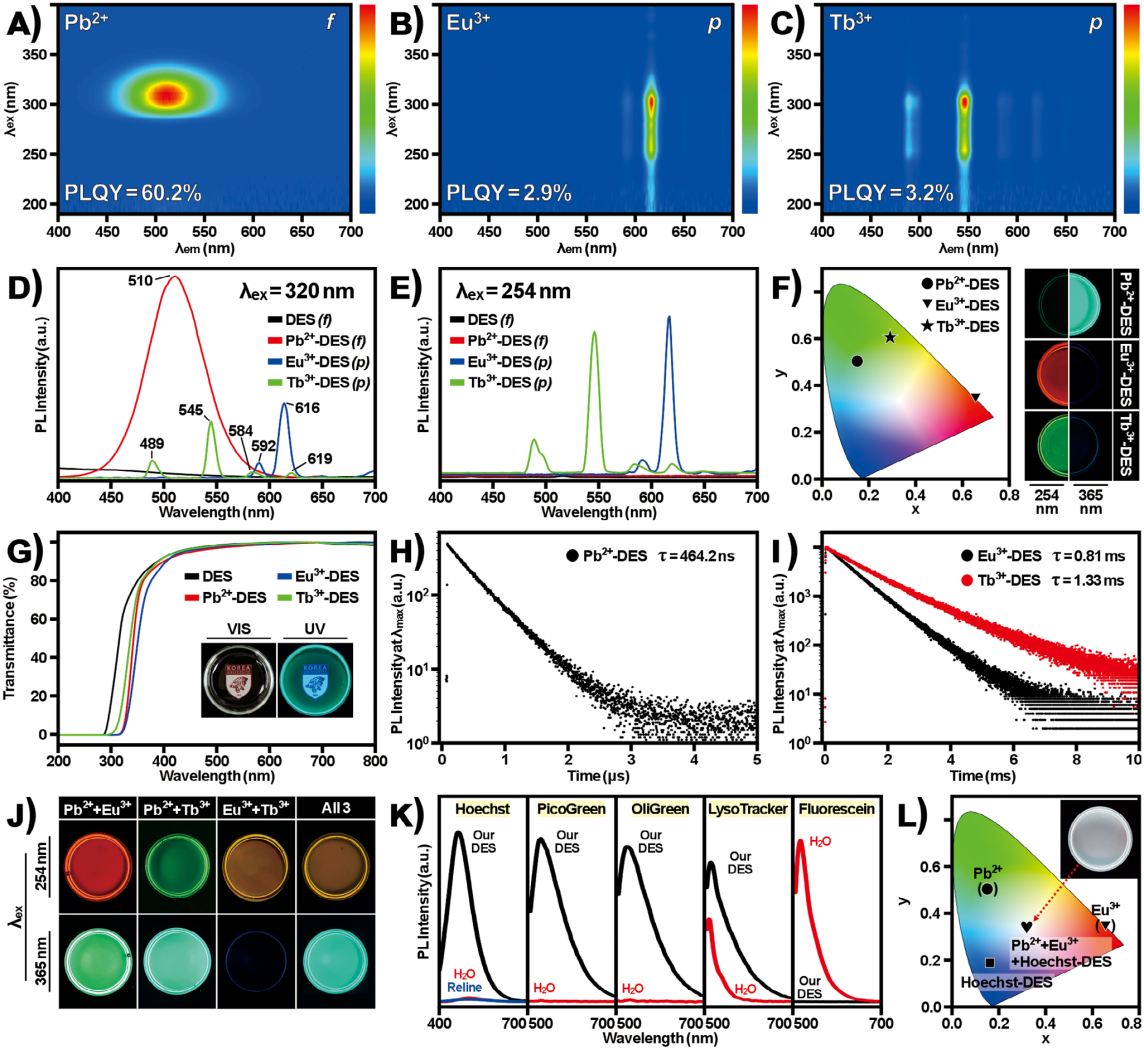
(A–C) Normalized EEM PL contour plots of BTEAC‐BA DESs doped with (A) Pb^2+^, (B) Eu^3+^, and (C) Tb^3+^, including their respective PLQY at 25°C. Note that the EEM for the Pb^2+^‐DES was acquired in fluorescence mode (*“f”*), whereas those of Eu^3+^‐ and Tb^3+^‐DESs were recorded in phosphorescence mode (*“p”*). (D and E) Representative PL spectra of the neat DES and M^n+^‐DESs recorded under (D) *λ_ex_* = 320 nm and (E) *λ_ex_* = 254 nm. (F) CIE 1931 chromaticity coordinates of M^n+^‐DESs and corresponding photographs under UV irradiation (*λ_ex_* = 254 and 365 nm). Note that the perceived colors under the UV lamps can differ slightly from those inferred from spectra collected under monochromatic excitation (e.g., 320 nm) because the lamps have different nominal wavelengths and finite spectral bandwidth. (G) UV‐vis transmittance spectra of the neat DES and M^n+^‐DESs (inset: photographs demonstrating the optical transparency of the Pb^2+^‐DES under visible light and 365 nm UV light). (H and I) TR‐PL decay curves of the (H) Pb^2+^‐DES (*λ_ex_* = 293 nm, *λ_em_* = 510 nm) and (I) Eu^3+^‐ and Tb^3+^‐DESs (*λ_ex_* = 300 nm, *λ_em_* = 616 nm for Eu^3+^ and 545 nm for Tb^3+^). (J) Photographs of multi‐cationic DES mixtures under UV irradiation (*λ_ex_* = 254 and 365 nm); “All 3” denotes the equivolumetric Pb^2+^+Eu^3+^+Tb^3+^‐DES combination. (K) PL spectra of diverse organic dyes dissolved in water and neat DESs under their respective optimized *λ_ex_*. (L) CIE 1931 chromaticity coordinates of the Hoechst‐DES and the Pb^2+^+Eu^3+^+Hoechst‐DES combination (inset: photograph of the Pb^2+^+Eu^3+^+Hoechst‐DES under 254 and 365 nm dual‐modal excitation).

The PL properties of the Pb^2+^‐doped DES (Pb^2+^‐DES) were first optimized as a function of Pb^2+^ loading (Figure S2). Emission intensity increased with Pb^2+^ concentration, plateauing at 0.05 mol% relative to BTEAC and BA; higher loadings resulted in precursor precipitation, establishing 0.05 mol% as the standard Pb^2+^ concentration. Excitation‐emission matrix (EEM) analysis identified a broad green emission centered at 510 nm (FWHM = 62 nm) under 320 nm excitation (Figure 2A). The PL quantum yield (PLQY) at this excitation reached 60.2%, placing this system among the most efficient non‐perovskite Pb^2+^ emitters reported as discrete coordination species [36, 43]. To assess the generality of the DES matrix for M^n+^‐induced PL, we further examined a set of M^n+^s spanning lanthanides (Ce^3+^, Nd^3+^, Eu^3+^, Gd^3+^, Tb^3+^, Dy^3+^, and Er^3+^), group 11/12 cations (Cu^+^, Cu^2+^, Ag^+^, and Au^3+^; Zn^2+^ and Cd^2+^), and several additional period 6 ions (Cs^+^, Ba^2+^, Ir^3+^, and Pt^2+^). PL was measured under 320 nm excitation, which optimally promotes Pb^2+^‐DES luminescence, and under higher energy 254 nm excitation, where Pb^2+^‐DES is largely inactive (Figure S3). Under these conditions, Eu^3+^, Tb^3+^, and Dy^3+^ exhibited characteristic lanthanide emissions, whereas the remaining M^n+^s remained hardly emissive. Owing to relatively weak emission of Dy^3+^, we selected Eu^3+^ and Tb^3+^, together with Pb^2+^, for detailed analysis. The neat DES displayed only weak broadband emission, confirming that the intense PL arises from metal incorporation. Under 320 nm excitation, Pb^2+^‐DES emitted strongly at 510 nm, whereas Eu^3+^‐ and Tb^3+^‐DESs showed narrow lanthanide bands with much lower PLQYs (Eu^3+^: 592 and 616 nm, 2.9%; Tb^3+^: 489, 545, 584, and 619 nm, 3.2%) (Figure 2B–E). Under 254 nm excitation, however, Eu^3+^‐ and Tb^3+^‐DESs became substantially brighter while Pb^2+^‐DES remained weak. CIE 1931 mapping yielded chromaticity coordinates of (0.15, 0.52) for Pb^2+^‐DES, (0.66, 0.33) for Eu^3+^‐DES, and (0.29, 0.63) for Tb^3+^‐DES (Figure 2F), demonstrating metal‐dependent color tunability. Photographs under 254 and 365 nm UV illumination further emphasized the excitation‐selective behavior of the M^n+^‐DESs. Under 254 nm, Pb^2+^‐DES was essentially dark, whereas Eu^3+^‐ and Tb^3+^‐DESs displayed bright red and green emission, respectively. Under 365 nm, Pb^2+^‐DES remained visibly emissive, while the lanthanide systems were largely off. This wavelength dependence is consistent with the EEM maps and establishes excitation wavelength and metal identity as orthogonal variables for selective optical readout. Despite their strong UV‐triggered emission, all M^n+^‐DESs remained highly transparent in the visible region, with transmittance close to 100% under white light (Figure 2G). UV–vis spectra showed that the neat DES absorbs strongly below 290 nm, whereas M^n+^ incorporation extends the absorption onset to ≈340 nm, consistent with efficient excitation of Pb^2+^‐DES at 320 nm. Time‐resolved PL further distinguished the emissive centers. Pb^2+^‐DES exhibited a sub‐microsecond lifetime of 464.2 ns at its emission maximum (Figure 2H), whereas Eu^3+^‐ and Tb^3+^‐DESs showed millisecond decays of 0.81 and 1.33 ms, respectively (Figure 2I), as expected for lanthanide‐centered 4f‐4f transitions [29, 52, 53]. This large lifetime contrast could offer an intrinsic time‐gating parameter, enabling temporally multiplexed information encoding. Equal‐volume mixtures of preformed M^n+^‐DESs broadened the accessible color space (Figure 2J). Mixtures of Pb^2+^‐DES with Eu^3+^‐DES or Tb^3+^‐DES produced dual‐modal outputs that appeared cyan under 365 nm excitation and red or green under 254 nm excitation, respectively. The Eu^3+^+Tb^3+^ mixture yielded bright yellow emission under 254 nm through additive color mixing. A ternary mixture containing Pb^2+^, Eu^3+^, and Tb^3+^ (All 3) combined dual‐modal and composite emission, whose EEM map was reproduced by linear superposition of the individual spectra (Figure S4). The absence of evident shifts, broadening, or quenching indicates minimal inter‐center coupling, which is advantageous for high‐density optical encoding with limited spectral crosstalk. The DES also accommodated organic fluorophores, further extending its chromatic range. Hoechst 33342, PicoGreen, OliGreen, LysoTracker, and fluorescein were evaluated in the BTEAC/BA matrix (Figure 2K). Hoechst showed strong PL enhancement in the DES, while remaining nearly nonemissive in water and in the conventional DES Reline (choline chloride/urea). This turn‐on is consistent with suppression of non‐radiative relaxation by restriction of intramolecular motion in the highly viscous DES, likely reinforced by aromatic interactions within the BTEAC/BA network [54]. Similar reasoning plausibly accounts for the enhanced emission of PicoGreen and OliGreen. LysoTracker also became markedly brighter, likely owing to favorable protonation within the BA‐rich microenvironment. In contrast, fluorescein was quenched owing to the concentrated quaternary ammonium environment [55]. Using the blue emission of Hoechst in the DES together with red‐emitting Eu^3+^‐DES and green‐emitting Pb^2+^‐DES, we prepared a white‐light‐emitting ternary system. Its CIE coordinates, (0.33, 0.34), are nearly identical to the standard white point (0.33, 0.33), highlighting the exceptional color expandability enabled by the DES host (Figure 2L).

### 2.3 Thermal, Photochemical, and Environmental Stability of M^n+^‐Doped DESs

**FIGURE 3:**
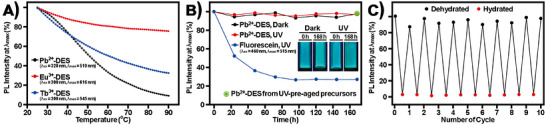
(A) Temperature‐dependent relative PL intensity profiles of the M^n+^‐DESs. (B) Photostability profiles of the Pb^2+^‐DES under dark conditions and continuous 365 nm UV irradiation, compared with an aqueous fluorescein reference. To evaluate the intrinsic stability of the DES components, an additional Pb^2+^‐DES was prepared using precursors that were individually pre‐aged under UV irradiation for 168 h. Inset: Photographs demonstrating the emission stability of Pb^2+^‐DES before and after 168 h of incubation under each condition. (C) Relative PL intensity profiles of Pb^2+^‐DES through 10 repetitive hydration‐dehydration cycles.

Thermal robustness of the M^n+^‐DESs was then examined (Figure 3A). Temperature‐dependent PL measurements, normalized at the respective emission maxima, showed a nearly monotonic decrease in intensity upon heating. At 90°C, Pb^2+^‐DES, Eu^3+^‐DES, and Tb^3+^‐DES retained 9.2%, 75.7%, and 32.6% of their initial emission, respectively. This thermal quenching can be attributed to reduced microviscosity and increased intermolecular separation at elevated temperature, leading to increased non‐radiative relaxation. The comparatively modest quenching of Eu^3+^‐DES is consistent with the parity‐forbidden and well‐shielded 4f transitions of Eu^3+^ and its reduced susceptibility to thermally activated back energy transfer relative to Tb^3+^ [56, 57]. Photostability was investigated using Pb^2+^‐DES as a representative example under 365 nm UV exposure and compared with that of aqueous fluorescein sodium salt (Figure 3B and Figure S5). Fluorescein lost approximately half of its initial intensity within 24 h of UV irradiation and then approached a plateau near 30%. In contrast, Pb^2+^‐DES exhibited negligible photobleaching even after 1 week. Its PL intensity remained comparable to that of an unexposed control sample consistent with the intrinsic photostability of BA and BTEAC (green dot, Figure 3B). The influence of hydration was explored by repetitive hydration‐dehydration cycling (Figure 3C). Adding water to Pb^2+^‐DES (DES:water = 2:1 v/v) nearly completely quenched the PL, far beyond the effect expected from simple dilution, likely because water molecules disrupt the DES hydrogen bond network and competitively hydrate Pb^2+^. Dehydration at 70°C for 12 h restored over 90% of the initial PL, and this reversible switching persisted over 10 cycles without substantial degradation. By contrast, addition of dichloromethane (DCM), a weakly hydrogen‐bonding solvent, caused a linear decrease in PL with DES fraction, indicating that the intrinsic emissive environment remained largely intact (Figure S6). Additionally, Pb^2+^‐DES was immiscible with toluene, forming a DES‐in‐oil emulsion in the presence of Triton X‐100, which underscores the highly polar and cohesive nature of the DES matrix (Figure S7). Together, these sharp contrasts show that the highly organized hydrogen bond architecture of the DES is essential for maintaining the emissive microenvironment. Placed in context, the M^n+^‐DES platform combines features that are rarely co‐located in conventional luminescent materials, including CsPbX_3_ perovskite nanocrystals, CdSe/ZnS and InP/ZnS quantum dots, and high‐performance organic dyes. A side‐by‐side comparison across cost, toxicity, and stability metrics is provided in Table S1. These unique properties attribute the M^n+^‐DES platform as a practically attractive complement to conventional emitters, particularly for applications in which device‐scale processing, operational lifetime, and ambient handling are as important as raw photophysical metrics.

### 2.4 Spectroscopic Elucidation of the DES Network and Pb^2+^ Coordination

**FIGURE 4:**
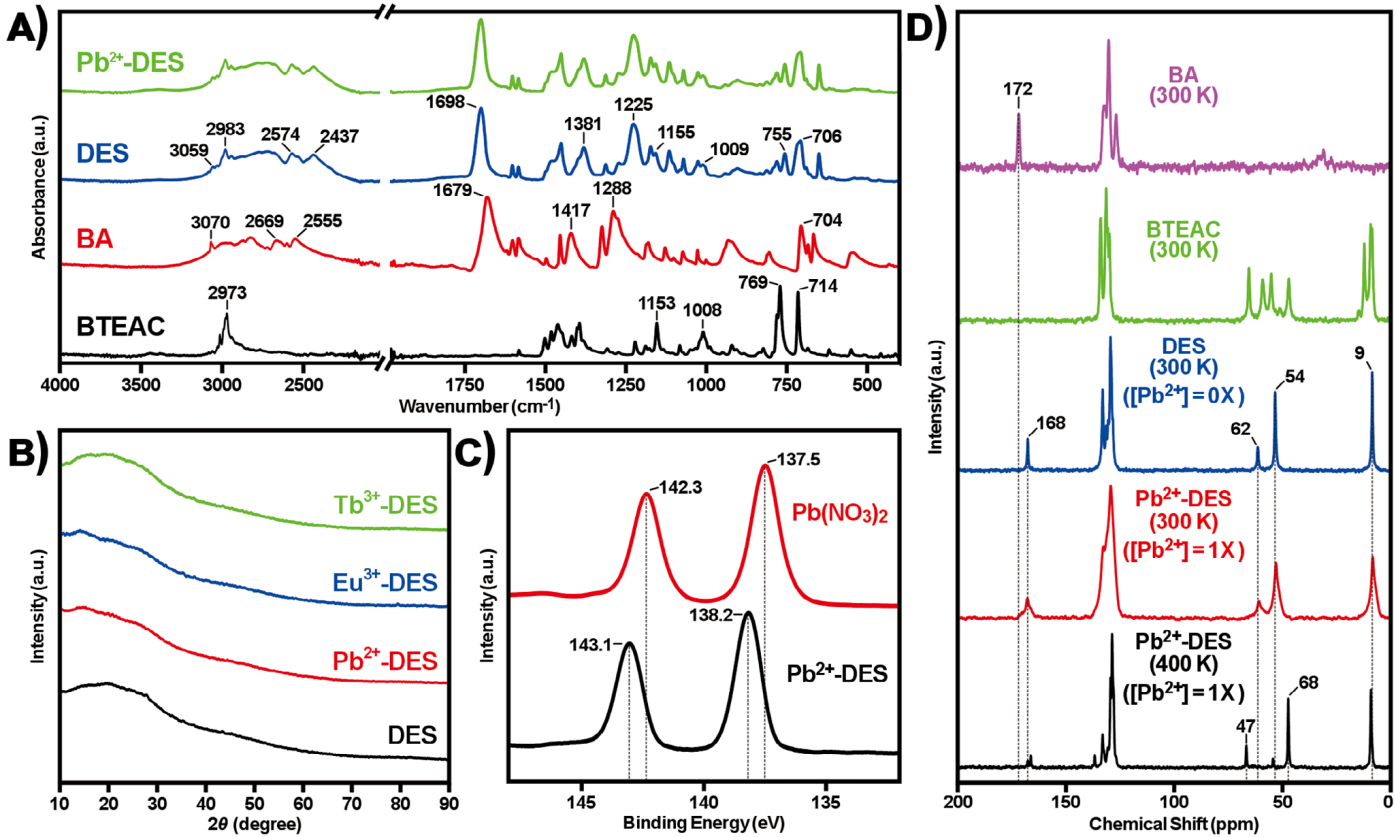
(A) FT‐IR spectra of the neat DES, Pb^2+^‐DES, and their constituent precursors (BA and BTEAC). (B) XRD profiles of the neat DES and M^n+^‐DESs. (C) XPS core‐level spectra of Pb 4f for the Pb^2+^‐DES and Pb(NO_3_)_2_ precursor. (D) Variable‐temperature solid‐state ^13^C NMR spectra of the Pb^2+^‐DES recorded at 300 and 400 K, compared with those of the neat DES and constituent precursors acquired at 300 K. Note that 1X corresponds to 0.05 mol% relative to BTEAC and BA.

To elucidate the chemical environment responsible for this behavior, we compared the Fourier‐transform infrared (FT‐IR) spectra of Pb^2+^‐DES with those of its constituent precursors (Figure 4A and Table S2). DES formation induced systematic shifts in BA‐derived vibrational modes to lower wavenumbers (O‐H stretching: 3070, 2669, and 2555 to 3059, 2574, and 2437 cm^−1^, respectively; O‐H bending: 1417 to 1381 cm^−1^; and C‐O stretching: 1288 to 1225 cm^−1^), consistent with establishment of an extensive intermolecular hydrogen bond network between BA and BTEAC [58, 59]. In contrast, the C = O stretching band shifted to higher wavenumber (1579 to 1698 cm^−1^), likely reflecting the inductive influence of Cl^−^ within the eutectic environment [60, 61]. A modest shift in the aromatic out‐of‐plane C‐H bending mode to higher wavenumber (704 to 706 cm^−1^) further suggested altered aromatic packing. Specifically, this shift suggests a closer *π*‐*π* association and the formation of non‐covalent aromatic networks (e.g., *π*‐*π* stacking and cation‐*π* interactions) within the DES environment, which possibly contributes to the stabilization of the emissive center. Given the low molar ratio of Pb^2+^ to BTEAC and BA (approximately 1:2000), no discernible change in the bulk FT‐IR profile was produced after Pb^2+^ addition, indicating that the macroscopic hydrogen bond framework is largely well preserved upon Pb^2+^ incorporation. X‐ray diffraction (XRD) patterns of the neat DES and M^n+^‐DESs contained only broad diffuse features with no sharp Bragg reflections, consistent with an amorphous liquid‐like phase without detectable crystalline precursor residues or long‐range‐ordered precipitates after M^n+^ addition (Figure 4B). Differential scanning calorimetry (DSC) thermograms of the neat DES and Pb^2+^‐DES showed glass transitions at ‐21.4 and ‐23.8°C and thermal decomposition peaks at 211.9 and 214.3°C, respectively, without crystalline melting transitions, confirming their broad operational window (Figure S8). X‐ray photoelectron spectroscopy (XPS) further probed the Pb coordination environment: the Pb 4f signals shifted from 142.3 and 137.5 eV in Pb(NO_3_)_2_ to 143.1 and 138.2 eV in Pb^2+^‐DES, indicating decreased electron density at the Pb center after incorporation and supporting coordination by electronegative donors such as Cl^−^ and oxygen‐containing species in the DES (Figure 4C). Solid‐state ^13^C nuclear magnetic resonance (NMR) spectroscopy further provided molecular‐level insight into local environments within the BTEAC/BA framework (Figure 4D and Table S3). Relative to crystalline BA, whose carboxyl carbon appears at *δ* 172 because of its dimeric O‐H···O = C hydrogen bond motif, the DES shows an upfield signal at *δ* 168, consistent with disruption of the BA dimer network and formation of a new hydrogen bond environment [62, 63]. BTEAC exhibits multiple nonaromatic resonances owing to crystallographic inequivalence and conformational freezing in solid state, but these collapse into a simpler, solution‐like pattern in the DES, indicating enhanced motional averaging [64, 65]. Upon Pb^2+^ addition, the ^13^C resonances of the DES broaden substantially without new well‐resolved peaks, implying a distribution of Pb‐perturbed local environments, although the low Pb^2+^ loading and carbon‐detected nature of the experiment do not exclude specific Pb‐associated species. At tenfold lower Pb^2+^ loading, much sharper resonances reappear (Figure S9), supporting a concentration‐dependent perturbation of the DES environments. Variable‐temperature ^13^C NMR experiments further support this interpretation: heating from 300 to 400 K markedly sharpens the resonances, consistent with motional narrowing. Thus, the NMR data indicate that Pb^2+^ introduces pronounced microheterogeneity and slow dynamic exchange within the amorphous DES, although the precise chemical identity of the Pb‐containing species cannot be assigned from ^13^C NMR alone.

### 2.5 MD and SOC‐TD‐DFT Studies on [PbCl_4_]^2−^ Confinement and Excited‐State Dynamics

**FIGURE 5:**
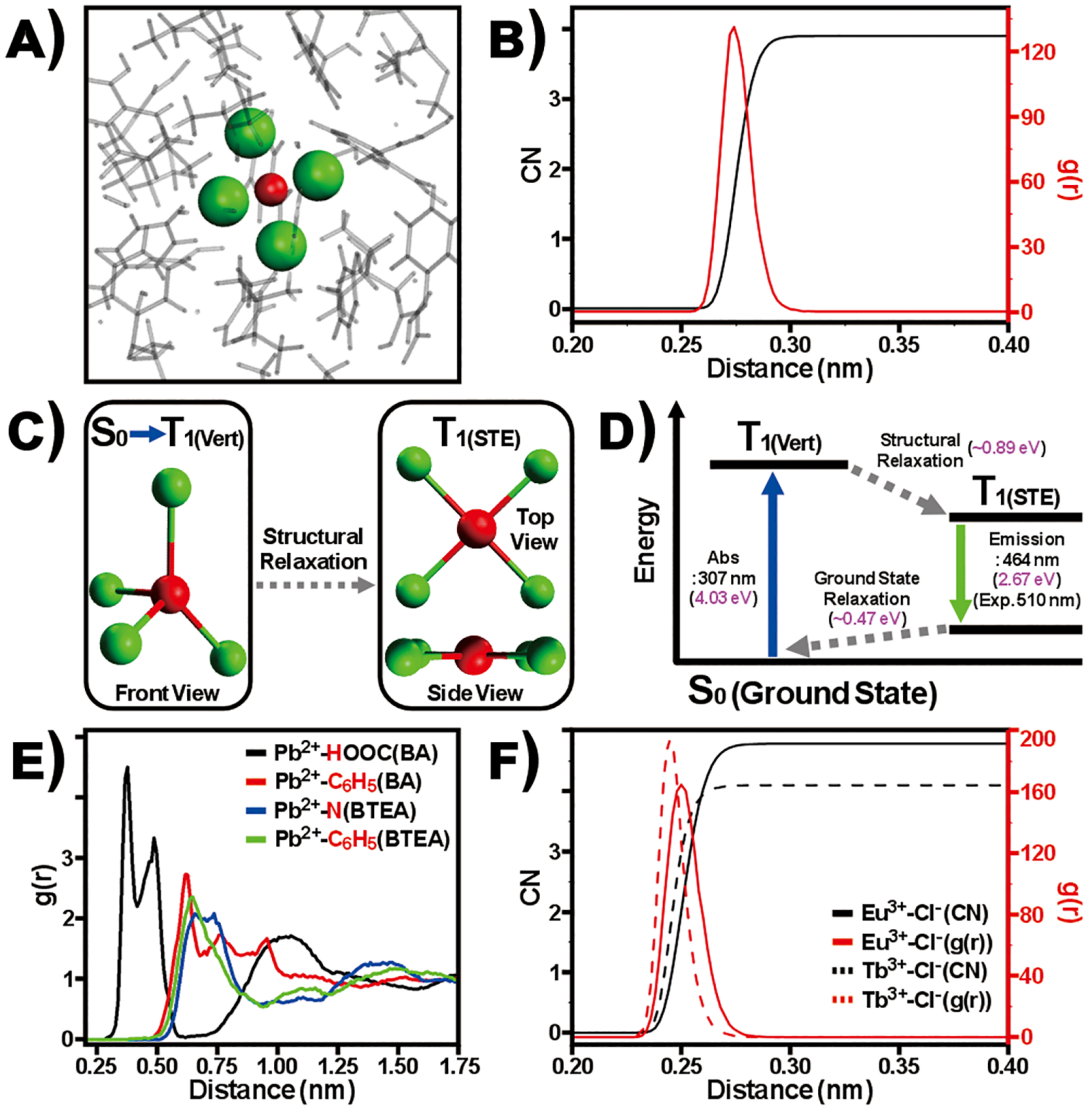
(A) A 10 ns MD simulation snapshot of distorted tetrahedral [PbCl_4_]^2−^ complex within the hydrogen bond network of BTEA^+^ and BA. Red sphere: Pb^2+^; green sphere: Cl^−^ (B) RDF (g(r), red curve) and the corresponding cumulative coordination number (CN, black curve) for the Pb‐Cl pairs. (C) DFT optimized structures of the extracted [PbCl_4_]^2−^ complex in the ground state (S_0_) and the lowest triplet excited‐state (T_1_). (D) Proposed Jablonski energy diagram illustrating the triplet STE emission mechanism. (E) RDFs illustrating the specific interactions between the Pb^2+^ center and various components of the DES (the acidic proton of BA (HOOC(BA)), the center of mass of BA benzene ring (C_6_H_5_(BA)), the nitrogen atom of BTEA^+^ (N(BTEA)), and the center of mass of BTEA^+^ benzene ring (C_6_H_5_(BTEA))). (F) RDFs (g(r), red curves) and CNs (black curves) for the lanthanide‐chloride interactions, specifically Eu^3+^‐Cl^−^ (solid lines) and Tb^3+^‐Cl^−^ (dashed lines) interactions in the DES.

To directly define the metal coordination motif, molecular dynamics (MD) simulations were performed at 353.15 K. An equilibrated trajectory snapshot revealed spontaneous formation of an isolated, 0D [PbCl_4_]^2−^ species in the DES without additional ligands (Figure 5A). This assignment is supported by the Pb‐Cl radial distribution function (RDF), which shows a pronounced first‐shell peak at 0.274 nm and integrates to a coordination number of 4.0 (Figure 5B). The resulting complex is markedly distorted relative to an ideal tetrahedron (Table S4), indicating that the hydrogen bond network and steric confinement of the DES shape the coordination geometry. Time‐dependent density functional theory calculations including spin‐orbit coupling (SOC‐TD‐DFT) rationalized the optical response, including the large Stokes shift. Experimentally, Pb^2+^‐ DES exhibits broad emission at ≈510 nm, an excitation maximum at 320 nm, and a sub‐microsecond lifetime of 464 ns, consistent with triplet self‐trapped exciton (STE) emission. The calculations show that the [PbCl_4_]^2−^ unit undergoes pronounced relaxation from a distorted tetrahedral‐like S_0_ geometry to a flatter T_1_ structure (Figure 5C), and the resulting photophysical cycle is summarized in the Jablonski diagram (Figure 5D). The calculated SOC‐allowed absorption at 307.4 nm agrees well with the experimental excitation maximum at 320 nm (Table S5), and the relaxation energy (≈0.89 eV) supports strong excited‐state self‐trapping. The calculated T_1_→S_0_ emission at 464 nm is close to the experimental 510 nm emission (*ΔE* ≈0.24 eV), reasonable for SOC‐TD‐DFT calculations on heavy‐metal complexes (Table S6). The remaining discrepancy likely arises from the simplified solvation treatment of the heterogeneous DES environment. The extremely small but non‐zero oscillator strength (*f* ≈4.9×10^−7^) is consistent with the measured sub‐microsecond lifetime. Taken together, the MD and SOC‐TD‐DFT results assign the efficient green emission to the triplet STE recombination from dynamically confined 0D [PbCl_4_]^2−^ complexes. Additional MD analysis clarified the surrounding solvation microenvironment (Figure 5E). Pb‐centered RDFs show that BA approaches the Pb‐containing center most closely through its carboxylic acid group, while BTEAC‐derived ammonium and aromatic moieties populate broader, more distant shells. This suggests that BA acidic groups preferentially stabilize the Cl^−^‐coordinated Pb center, while the bulky cations create a sterically crowded outer environment. Beyond the carboxylate‐mediated hydrogen bonds to chloride, the same trajectory reveals a structured aromatic sub‐network around the [PbCl_4_]^2−^ cage. Centroid‐centroid RDFs computed for the ring pairs show pronounced first‐shell features (Figure S10). Specifically, first‐shell peaks near 0.45 nm and 0.49 nm (as observed for BTEA‐BTEA and BA‐BTEA pairs, respectively) are the canonical fingerprint of parallel‐offset π‐π stacking, whereas the prominent peak centered at ≈0.55 nm (observed for BA‐BA pairs) typically indicates a T‐shaped geometry. Cation‐π contacts are equally well populated, with the N^+^(BTEA)‐C_6_H_5_(BA) RDF displaying a sharp peak near 0.50 nm. Together, these structural features identify a highly organized, layered solvation architecture. Although energetically smaller than the hydrogen bond contribution, these aromatic interactions geometrically rigidify the outer shell, effectively suppressing non‐radiative decay pathways and constituting a previously underappreciated handle for fine‐tuning the emissive [PbCl_4_]^2−^ center. Parallel simulations with lanthanides show that the same DES microenvironment imposes coordination constraints that depend sensitively on ionic radius (Figure 5F). Both Eu^3+^ and Tb^3+^ exhibited first‐shell Ln‐Cl peaks near 0.25 nm, but their Cl^−^ coordination number differed (≈4.8 for Eu^3+^ and ≈4.1 for Tb^3+^), consistent with lanthanide contraction and a slightly tighter chloride environment around Tb^3+^. As with Pb^2+^, these in situ formed chloride complexes are strongly implicated as the primary emissive centers in the lanthanide DESs [66, 67].

### 2.6 Structural Determinants of Halide Speciation and Emissive Microenvironments

**FIGURE 6:**
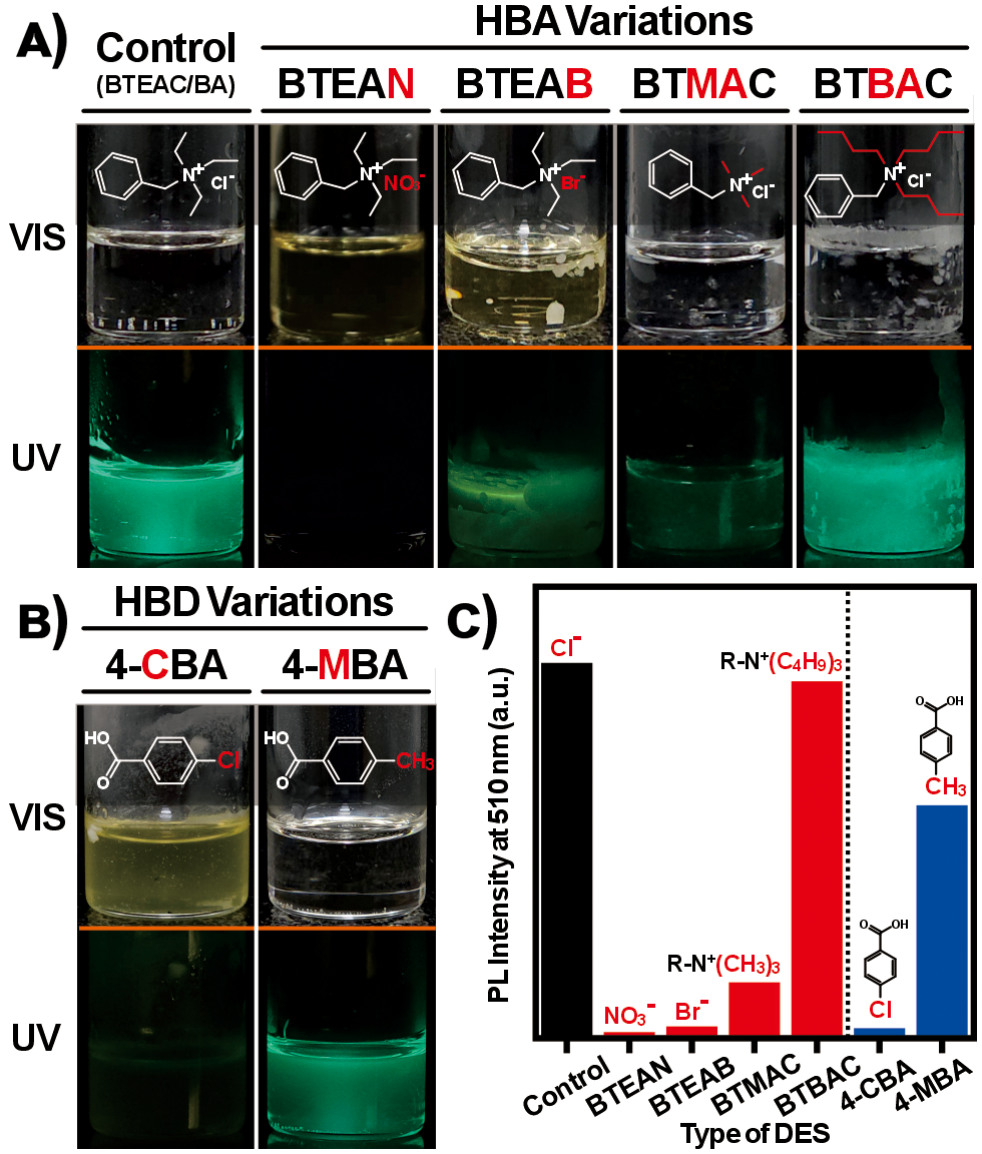
(A and B) Visual observation of Pb^2+^‐DES series used for the experimental validation of MD and DFT predictions. Photographs show combinations of (A) BA with varying HBAs and (B) BTEAC with varying HBDs. Top rows: under visible light (VIS); bottom rows: under UV irradiation (*λ_ex_* = 365 nm). (C) Comparison of PL intensities at 510 nm for the Pb^2+^‐DES series acquired under *λ_ex_* = 320 nm.

Guided by the screening results and mechanicstic modeling above, a targeted study using structurally controlled BTEAC and BA derivatives further elucidated the origin of exceptional PL in the BTEAC/BA system and the identity of the emissive Pb species (Figure 6). Under visible light, all compositions appeared transparent and either colorless or faintly yellow, but their UV responses depended strongly on HBA and HBD structure (Figure 6A,B). Replacing Cl^−^ in BTEAC with NO_3_^−^ (BTEAN) abolished the PL, whereas substitution with Br^−^ (BTEAB) produced only weak emission. Combined with the MD‐derived chloride‐rich coordination motif, these results demonstrate that chloride is indispensable for formation of the efficient emitter and support assignment to 0D chloroplumbates, rather than uncoordinated Pb^2+^ or chloride‐free Pb complexes. Mixed‐halide experiments further strengthened this rationale: progressive replacing Cl^−^ with Br^−^ in the HBA component monotonically decreased the PLQY to around 4% and shortened the PL lifetime to 17 ns (Figure S11). Such systematic photophysical changes indicate modulation of the emissive center itself, likely through formation of mixed‐halide [PbCl_4−x_Br_x_]^2−^ species. Furthermore, Raman spectroscopy of the Pb^2+^‐DES displayed a distinct band at 231 cm^−1^ absent from the neat DES, characteristic of Pb‐Cl vibrations in chloroplumbate(II) species (Figure S12) [68, 69, 70]. The cationic environment also proved critical: replacing the ethyl substituents of BTEAC with methyl groups (BTMAC) severely weakened the PL, whereas bulkier butyl groups (BTBAC) preserved emission comparable to the control. This suggests that the quaternary ammonium cation contributes to a protective microenvironment around the anionic emitter under appropriate steric separation. Substituent effects were also significant on the HBD side. Replacing BA with 4‐chlorobenzoic acid (4‐CBA) nearly eliminated emission, whereas 4‐methylbenzoic acid (4‐MBA) retained moderate emission. Because BA interacts most strongly with the Pb center through its carboxylic acid group in the simulations, these results suggest that para substitution perturbs the local hydrogen‐bonding environment that stabilizes the emissive species. Although the corresponding BTEAC/4‐CBA system exhibits increased absorbance in the UV and visible regions contributing to reabsorption of the PL (Figure S13), this optical effect alone is unlikely to explain the near‐complete quenching. Rather, the electron‐withdrawing Cl substituent likely alters acidity and polarization sufficiently to disrupt the optimal local environment, whereas the electron‐donating methyl substituent in 4‐MBA is less disruptive. The corresponding PL spectra and normalized intensities at 510 nm confirm these trends: BTEAN/BA and BTEAC/4‐CBA were essentially non‐emissive, BTEAB/BA and BTMAC/BA were weakly emissive, BTEAC/4‐MBA retained approximately 60% of the control intensity, and BTBAC/BA remained nearly as bright as BTEAC/BA (Figure 6C and Figure S14). The structural perturbation series can now be interpreted in light of the aromatic sub‐network identified above. Sensitivity of the PL intensity to the alkyl‐chain length on BTEAC is modest (BTMAC ≤ BTBAC ≈ BTEAC), whereas substitution of the BA ring at the para position causes substantial changes (4‐CBA <<< BA). This asymmetric response is consistent with a configuration in which the BA phenyl ring participates directly in *π‐π* and cation‐*π* contacts that shape the outer coordination sphere of [PbCl_4_]^2−^, while the BTEA^+^ alkyl arms primarily provide steric bulk and play a secondary role in aromatic packing. Electron‐withdrawing chlorination of the BA ring weakens *π*‐electron density, whereas the mildly electron‐donating methyl group preserves them. The resulting picture, in which steric, hydrogen bond, and aromatic interactions act on distinct shells, rationalizes the synergistic performance of the BTEAC/BA pair.

### 2.7 Translation Into Photoluminescent Eutectogels With Tunable Mechanics

**FIGURE 7:**
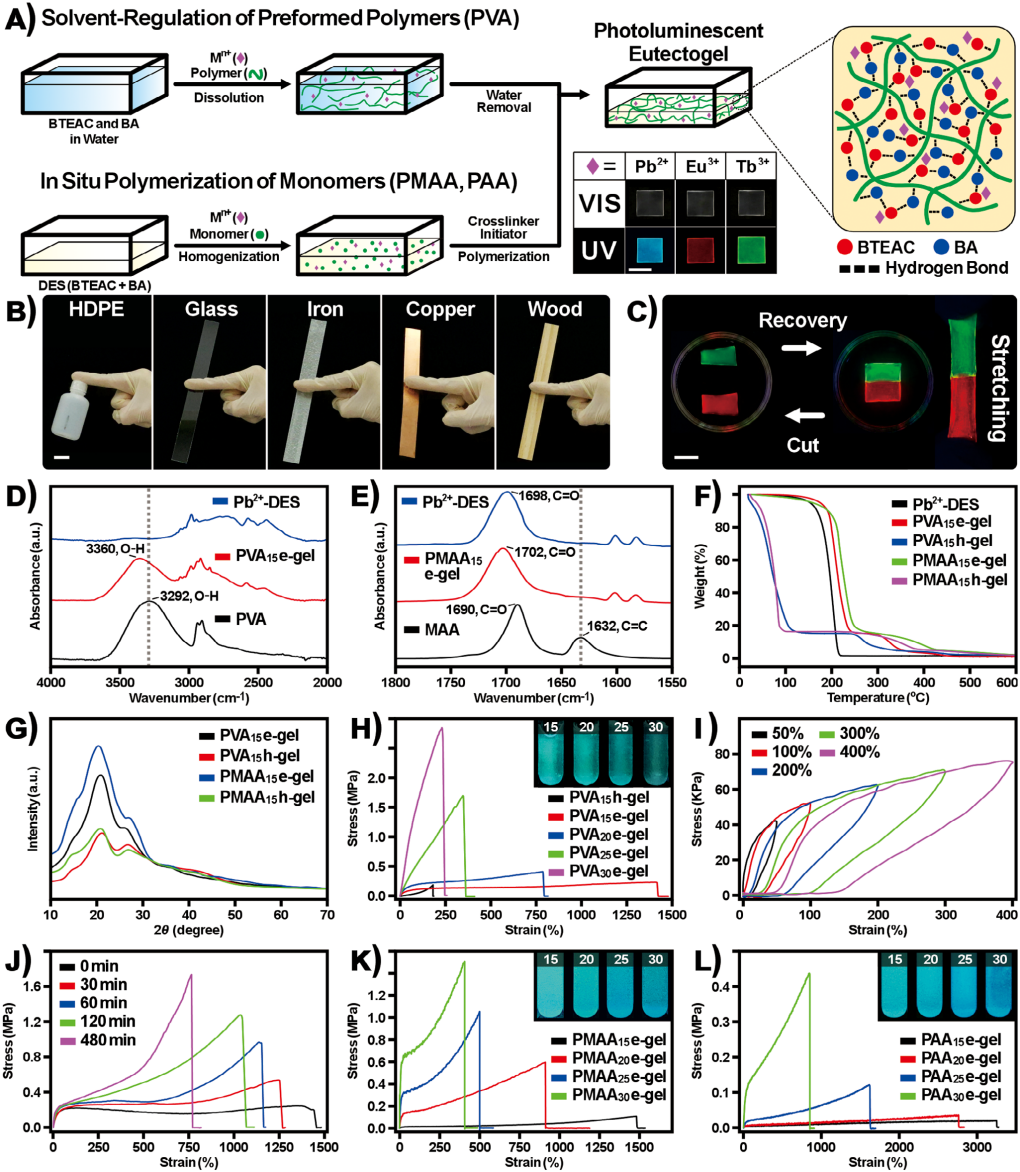
(A) Schematic illustration of the M^n+^‐DES‐based eutectogel synthesis via solvent regulation and in situ polymerization with the underlying gel strengthening mechanism. (B and C) Photographs demonstrating the (B) versatile adhesion of the PVA_15_ eutectogel to diverse substrate surfaces (high‐density polyethylene (HDPE), glass, iron, copper, and wood) and (C) its intrinsic self‐healing capability. (D and E) FT‐IR spectra of (D) PVA_15_ and (E) PMAA_15_ eutectogels compared to their respective precursors. Note that eutectogel and hydrogel are abbreviated as e‐gel and h‐gel, respectively. (F) XRD patterns of PVA_15_ and PMAA_15_ e‐gels. (G) TGA profiles comparing the thermal stability of Pb^2+^‐DES, e‐gels, and h‐gels. (H) Tensile stress‐strain curves of PVA e‐gels as a function of polymer content (inset: photographs of corresponding luminescent e‐gels under 365 nm UV irradiation). (I) Hysteresis behavior of the PVA_15_ e‐gel characterized by cyclic loading‐unloading tests at incremental strains of 50%–400%. (J) Tensile stress‐strain curves of PVA_15_ e‐gels after additional drying for different durations. (K and L) Tensile stress‐strain curves of (K) PMAA and (L) PAA e‐gels as a function of polymer content (insets: photographs of corresponding luminescent e‐gels under 365 nm UV irradiation). Scale bars represent 1 cm.

We next translated the M^n+^‐DESs into mechanically robust soft materials by incorporating them into polymer networks to form photoluminescent eutectogels. To demonstrate generality, two synthetic routes were implemented: a solvent‐regulation strategy using preformed polymers and in situ polymerization strategy using monomers (Figure 7A). The former is advantageous for polymers such as poly(vinyl alcohol) (PVA), for which direct monomer‐based synthesis is impractical, whereas the latter offers a direct route for monomers such as methacrylic acid (MAA) [71]. PVA and poly(methacrylic acid) (PMAA) were therefore selected as representative systems, and the resulting materials are denoted PVA_15_ to PVA_30_ and PMAA_15_ to PMAA_30_ eutectogels according to polymer content. The PVA_15_ eutectogel exhibited strong adhesion to diverse substrates, including HDPE, glass, iron, copper, and wood (Figure 7B), consistent with the amphiphilic nature of the DES, supported by a contact angle study demonstrating superior wettability of the DES across varied surface polarities (Figure S15). The same eutectogel also displayed efficient self‐healing, attributable to reversible non‐covalent interactions between the DES and polymer chains (Figure 7C) [72]. Mechanical reinforcement relative to conventional hydrogels was substantial: a 1 cm thick PVA_15_ eutectogel supported a 1.5 kg load without rupture, whereas a hydrogel analogue failed under identical conditions (Figure S16). FT‐IR was used to characterize the molecular organization of the eutectogels (Figure 7D). In the PVA series, the O‐H stretching band shifted from 3292 cm^−1^ in the pristine PVA to 3360 cm^−1^ in the PVA_15_ eutectogel, indicating disruption of PVA‐PVA hydrogen bonds upon dissolution in the DES and formation of a more homogeneous network where hydroxyl groups vibrate more freely. In the PMAA system, the C = C stretching band of the MAA monomer at 1632 cm^−1^ disappeared after gelation, confirming effective in situ polymerization, while the C = O band shifted from 1690 cm^−1^ to 1702 cm^−1^, consistent with the BA‐rich environment (Figure 7E). Furthermore, analysis of the higher wavenumber region revealed a shift in the O‐H stretching band from 2965 cm^−1^ to 3408 cm^−1^ upon MAA polymerization and eutectogel formation (Figure S17). These consistent spectral shifts across both the PVA and PMAA systems demonstrate that the profound integration of the DES disrupts the inherent polymeric interchain bonds, replacing them with a dense, dynamic network of DES‐polymer interactions. Thermogravimetric analysis (TGA) confirmed the DES‐induced thermal stability of the eutectogels (Figure 7F). Unlike their hydrogel counterparts, which fully dehydrated near 100°C, PVA_15_ and PMAA_15_ eutectogels exhibited only 5% mass loss up to 175°C and 159°C, respectively. Subsequent mass loss (≈85%) aligned with thermal degradation of Pb^2+^‐DES. Notably, the polymeric frameworks remained stable up to 283°C (PVA_15_) and 275°C (PMAA_15_), demonstrating their viability for high‐temperature applications. Additionally, minimal residual water in the PVA_15_ eutectogel TGA profile confirmed a highly effective solvent‐regulation process. XRD was utilized to evaluate the crystalline organization of the eutectogels (Figure 7G). XRD patterns of the PVA_15_ and PMAA_15_ eutectogels exhibited a pronounced increase in the diffraction feature at 2θ = 20.3° relative to the corresponding hydrogels, indicating that the DES promotes a higher degree of gel crystallinity. These induced crystalline domains likely act as physical cross‐linking nodes that distribute stress and dissipate energy during deformation [73, 74]. Based on this structural rationale, we further evaluated the tensile and PL properties of the PVA eutectogels as a function of polymer content (Figure 7H, Figure S18 and Table S7). A conventional PVA_15_ hydrogel prepared by freeze‐thawing exhibited a tensile modulus of 0.04 MPa and a toughness of 0.11 MJ m^−3^, whereas PVA_15_ eutectogel reached 0.41 MPa and 2.38 MJ m^−3^, corresponding to approximately 10‐fold and 22‐fold enhancements, respectively. These improvements highlight the stronger solvent‐polymer interactions and the resulting mechanical reinforcement imparted by the M^n+^‐DES relative to water. Crucially, the self‐healed PVA_15_ eutectogel retained 93% of its original modulus and 74% of its toughness, demonstrating efficient structural recovery through the dynamic hydrogen bond network (Figure S19). Increasing the PVA content up to 30 wt% further raised the tensile modulus and toughness to 1.65 MPa and 4.18 MJ m^−3^, respectively, owing to denser polymer networks. However, this also reduced elongation at break and PL intensity, because the relative fraction of M^n+^‐DES decreased. Cyclic loading‐unloading measurements at varying strains revealed pronounced hysteresis and efficient energy dissipation in PVA_15_ eutectogel (Figure 7I). As the applied strain increased from 50% to 400% without intervals, the dissipated energy per cycle rose from 9.93 to 109.63 kJ∙m^−3^. The corresponding energy‐dissipation ratio was 61.4%–55.0%, respectively. Taken together, the superior mechanical properties of the eutectogels are governed by a synergistic, multi‐modal reinforcement mechanism. While the DES‐induced crystalline domains act as physical cross‐linking nodes, the fundamental toughening originates from the active remodeling of the internal hydrogen bond network. The dense array of non‐covalent DES‐polymer interactions likely increases the overall cohesive energy of the matrix, tightly holding the polymer chains together in response to mechanical stimuli. Furthermore, the BTEAC/BA system introduces a continuous spectrum of interaction strengths, ranging from weak dipole and aromatic contacts to strong ion‐mediated crosslinks. Under tensile load, the weaker bonds reversibly rupture to dissipate energy, whereas the stronger interactions preserve network connectivity. Consequently, as evidenced by the pronounced hysteresis, these bonds serve as sacrificial linkages that dissipate applied strain energy during deformation, preventing macroscopic failure and endowing the materials with exceptional toughness. Post‐synthetic thermal vacuum treatment provided additional route to mechanical reinforcement (Figure 7J and Table S8). Heating PVA_15_ eutectogel at 100°C under vacuum progressively increased the tensile modulus and toughness from 0.41 MPa and 2.38 MJ m^−3^ to 1.72 MPa and 6.44 MJ m^−3^, respectively, likely because removal of trapped bubbles and trace amount of residual water reduced structural defects and enabled closer DES‐polymer contact. To test whether these trends are general across polymer backbones and synthetic routes, we further analyzed in situ polymerized PMAA and additionally poly(acrylic acid) (PAA) eutectogels as a function of polymer content (Figure 7K,L, and Figure S18). In both systems, tensile strength and toughness increased with increasing polymer content, from 0.09 MPa and 0.60 MJ m^−3^ to 5.47 MPa and 6.44 MJ m^−3^ in the PMAA series and from 0.03 MPa and 0.45 MJ m^−3^ to 0.49 MPa and 2.18 MJ m^−3^ in the PAA series, respectively. Correspondingly, the consistent trade‐off between mechanical reinforcement and optical output applied, confirming that M^n+^‐DES incorporation offers a modular strategy for preparing luminescent soft materials with tunable mechanics.

### 2.8 Multilevel Information Encoding and Anti‐Counterfeiting Applications

**FIGURE 8:**
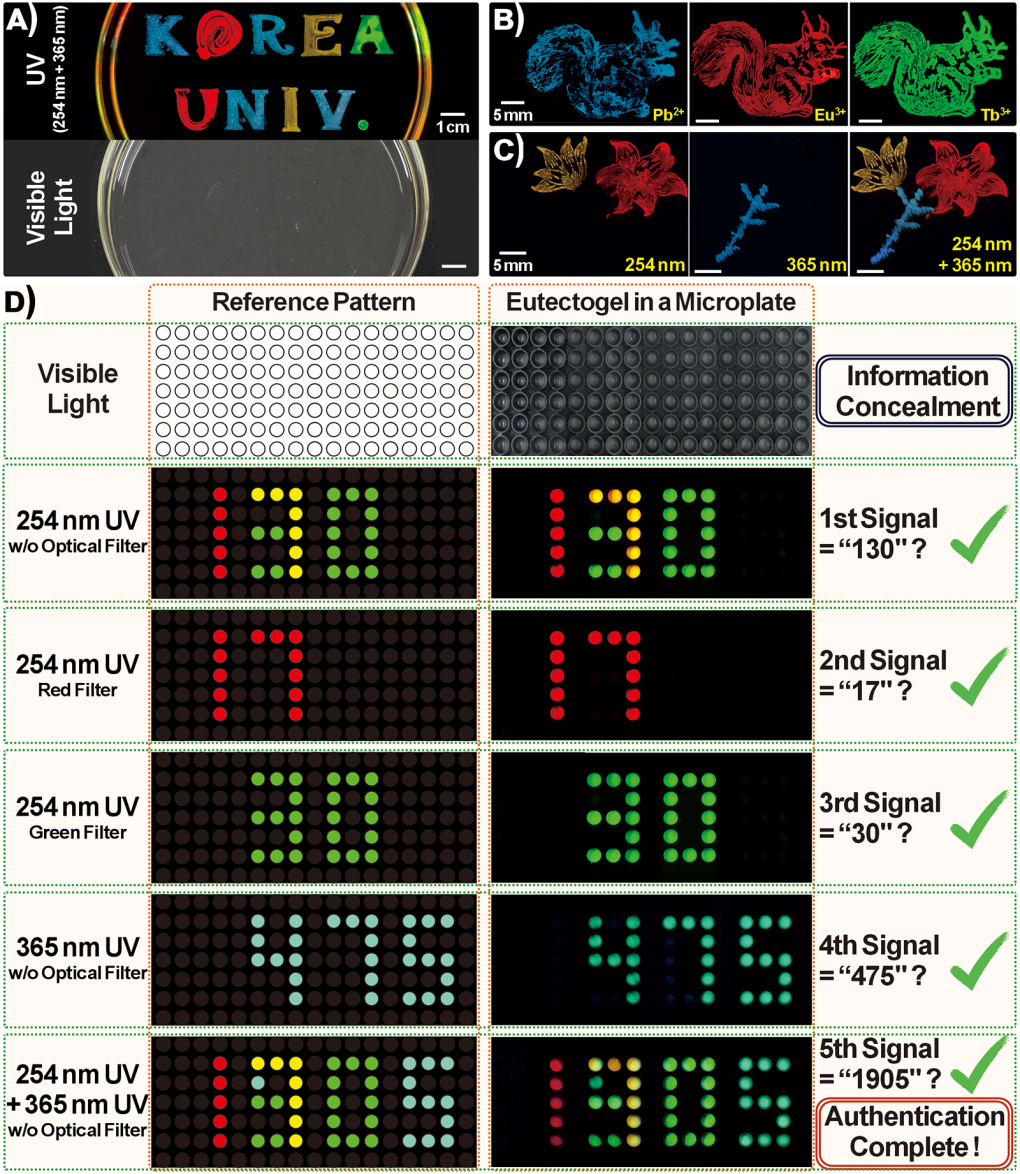
(A) Photographs of the alphabetic pattern “KOREA UNIV.” encrypted via the stamping of aqueous M^n+^ inks onto a PVA eutectogel “blank canvas.” Top: under dual‐wavelength UV irradiation (*λ_ex_* = 254/365 nm); bottom: under visible light. Consonants were rendered using Pb^2+^; ‘O’ and ‘U’ with Eu^3+^; ‘A’ and the period mark with Tb^3+^; and ‘E’ and ‘I’ with a combination of Eu^3+^ and Tb^3+^. (B) High‐resolution graphical motifs of squirrels encrypted using individual Pb^2+^ (*λ_ex_* = 365 nm), Eu^3+^ (*λ_ex_* = 254 nm), and Tb^3+^ (*λ_ex_* = 254 nm) solutions, observed under their respective UV irradiation. (C) Wavelength‐selective decryption of a composite flower pattern. Petals were encrypted with Eu^3+^ (red) and a combination of Eu^3+^ and Tb^3+^ (yellow), while the stem was rendered with Pb^2+^. (D) Schematic and experimental demonstration of a hierarchical authentication process using a microplate with spatially arranged PVA eutectogels. The system utilizes sequential excitation (254 nm, 365 nm, and dual‐mode UV irradiation) and narrow‐bandpass optical filtering to reveal discrete numerical passcodes (“130”, “17”, “30”, “475”, and “1905”) required for the final authentication.

Luminescent multilevel systems are attractive for security applications because their outputs can be switched among multiple states in response to external stimuli, making them difficult to replicate and able to store dense information within a compact format [75, 76, 77, 78, 79, 80]. The present DES platform is well suited to this purpose owing to their excitation‐wavelength‐dependent and M^n+^‐specific emission signatures. Graphical information was first encoded by stamping aqueous M^n+^ solutions onto a neat PVA eutectogel “blank canvas” (Figure S20). As shown in Figure 8A, the pattern “KOREA UNIV.” was rendered with vivid and M^n+^‐specific emission colors while maintaining high spatial resolution. Under visible light, the same substrate remained transparent and featureless, effectively concealing the encoded information. More complex motifs, including squirrels and flowers, were similarly rendered with fine structural detail (Figure 8B,C). The flower pattern further demonstrated dual‐stimulus decryption: the petals and stem were designed to respond selectively to 254 and 365 nm excitation, respectively, so the complete silhouette emerged only under combined dual‐wavelength illumination. We then extended this concept to a microplate‐based multilevel authentication system built from spatially arranged M^n+^‐DES eutectogels (Figure 8D). Because the emissions of the individual channels combine linearly, successful decryption requires both the correct excitation wavelength and the appropriate emission filter. In the absence of UV irradiation, the plate remained visually inactive. Under 254 nm irradiation, however, a composite signal, “130,” appeared; using red and green band‐pass filters, this signal could be decomposed into the hidden components “17” and “30,” respectively. Switching to 365 nm excitation revealed an additional hidden signal, “475,” originating from Pb^2+^‐DES. Simultaneous irradiation at 254 and 365 nm generated the terminal passcode, “1905.” This hierarchy illustrates how independent emissive channels can be linearly superposed to create a compact but difficult‐to‐replicate verification scheme. Because the number of accessible color combinations grows rapidly with the number of individual primary channels, further incorporation of additional M^n+^s or organic fluorophores should substantially expand the complexity and security level of the platform for advanced anti‐counterfeiting applications.

## 3 Conclusion

We synthesized a highly photostable, M^n+^‐responsive photoluminescent DES platform through combinatorial screening of HBA/HBD libraries. These DESs exhibit M^n+^‐tunable, multi‐color emission characterized by distinctly customizable excitation and emission wavelengths, PL lifetimes, and PLQYs (reaching 60.2%) without requiring ex situ‐synthesized luminophores. Spectroscopic analyses and computations collectively indicate that the emission originates from in situ generated 0D halometallates, exemplified by [PbCl_4_]^2−^ center uniquely stabilized by the dynamic DES microenvironment. Integration of these DESs into polymer networks yielded mechanically robust, photoluminescent eutectogels that preserved the optical functionality of the DES while gaining the processability and strength of soft solids. The independent emission channels of these materials enabled facile construction of a high‐fidelity, multi‐level anti‐counterfeiting platform. Importantly, this work fundamentally redefines the utility of nonconventional liquids, including DESs. Beyond serving as passive solvent media, DESs can act as highly dynamic, functional matrices that directly govern dopant coordination and speciation, enabling stabilization of complexes that are hardly accessible in conventional aqueous, organic, or polymeric environments. Given their exceptional compositional tunability, we expect that DESs would further provide extensive opportunities to discover emergent properties of materials previously obscured by conventional solvent environments, promoting significant advancements in photonics, catalysis, and supramolecular chemistry for imaging, sensing, and biomedical applications [71].

## Author Contributions

**Jaeyune Ryu**: investigation, writing – original draft, writing – review and editing, conceptualization, methodology, supervision. **Woojin Jung**: investigation. **Minkyu Jung**: investigation. **Jae‐Seung Lee**: conceptualization, investigation, writing – original draft, writing – review and editing, methodology, software, supervision. **Jeesu Moon**: conceptualization, methodology, writing – review and editing, writing – original draft, investigation, software.

## Funding

This work was supported by National Research Foundation of Korea (NRF) grants funded by the Korea government (MSIT) (RS‐2026‐25482415) and the Korea government (MOE) through the BK21 FOUR Program (4199990514635).

## Conflicts of Interest

The authors declare no conflicts of interest.

## Supporting information




**Supporting File**: advs76012‐sup‐0001‐SuppMat.pdf.

## Data Availability

The data that supports the findings of this study are available in the supplementary material of this article.
