# Supplementary material for: In Situ Confinement of 0D Halometallates Within Deep Eutectic Solvents: From Systematic Screening to Metal‐Tunable Luminescence for Anti‐Counterfeiting Eutectogels
Source: Adv Sci (Weinh). 2026 Jun 4;13(48):e76012. doi: 10.1002/advs.76012 (PMC13336794; doi:10.1002/advs.76012)
Supplement: Supplementary file 1 [file ADVS-13-e76012-s001.pdf]

Supporting Information

In Situ Confinement of 0D Halometallates within Deep Eutectic Solvents: From Systematic Screening to Metal-Tunable Luminescence for Anti-Counterfeiting Eutectogels

Jeesu Moon[a], Minkyu Jung[a], Woojin Jung[b], Jaeyune Ryu[b] and Jae-Seung Lee*[a]*

[a] J. Moon, M. Jung, J.-S. Lee
Department of Materials Science and Engineering
Korea University
145 Anam-ro, Seongbuk-gu, Seoul 02841, Republic of Korea
E-mail: jslee79@korea.ac.kr

[b] W. Jung, J. Ryu
School of Chemical and Biological Engineering, Institute of Chemical Processes
Seoul National University
1 Gwanak-ro, Gwanak-gu, Seoul 08826, Republic of Korea
E-mail: jaeyune.ryu@snu.ac.kr

1. Experimental Section

1.1. Materials. $Pb(NO_3)_2$ (cat. no. 228621), $Eu(NO_3)_3·5H_2O$ (cat. no. 207918), $Tb(NO_3)_3·5H_2O$ (cat. no. 325945), CuCl (cat. no. 229628), $CuCl_2$ (cat. no. 751944), $ZnCl_2$ (cat. no. 793523), $AgNO_3$ (cat. no. 204390), $CdCl_2$ (cat. no. 20899), CsCl (cat. no. 289329), $Ba(NO_3)_2$ (cat. no. 217581), $Ce(NO_3)_3·6H_2O$ (cat. no. 392219), $Nd(NO_3)_3·6H_2O$ (cat. no. 289175), $Gd(NO_3)_3·6H_2O$ (cat. no. 451134), $Dy(NO_3)_3·xH_2O$ (cat. no. 298158), $Er(NO_3)_3·5H_2O$ (cat. no. 298166), $IrCl_3·xH_2O$ (cat. no. 203491), K_2PtCl_4 (cat. no. 323411), $HAuCl_4·3H_2O$ (cat. no. 520918), choline chloride (ChCl, cat. no. C1879), betaine (cat. no. B2629), tetraethylammonium chloride monohydrate (cat. no. 86605), glycine (cat. no. 50046), L-proline (cat. no. P5607), L-histidine (cat. no. 53319), nicotinic acid (cat. no. N4126), urea (cat. no. U5378), acetamide (cat. no. A0500), oxalic acid (cat. no. 75688), malonic acid (cat. no. 792535), succinic acid (cat.no. 398055), citric acid (cat. no. 251275), benzoic acid (BA, cat. no. 242381), 4-chlorobenzoic acid (4-CBA, cat. no. 135585), 4-methylbenzoic acid (4-MBA, cat. no. T36803), bisbenzimide H 33342 trihydrochloride (Hoechst, cat. no. B2261), fluorescein sodium salt (cat. no. 46960), dichloromethane (DCM, cat. no. 66749), poly(vinyl alcohol) (PVA, M.W. = 89,000 ~ 98,000, cat. no. 341584), acrylic acid (AA, cat. no. 147230), 2-hydroxyethyl acrylate (HEA, cat. no. 292818), methacrylic acid (MAA, cat. no. 155721), *N,N'*-methylenebisacrylamide (cat. no. M7279), toluene (cat. no. 244511), Triton X-100 (cat. no. X100), and $K_2S_2O_8$ (cat. no. 216224) were purchased from Merck KGaA (Darmstadt, Germany). Trimethylphenylammonium chloride (cat. no. P0244), benzyltrimethylammonium chloride (BTMAC, cat. no. B0447), benzyltriethylammonium chloride (BTEAC, cat. no. B0444), benzyltriethylammonium bromide (BTEAB, cat. no. B0443), benzyltributylammonium chloride (BTBAC, cat. no. B1384), benzoylcholine chloride (BChCl, cat. no. B0107), methyltriphenylphosphonium bromide (cat. no. M0779), 1-methylurea (cat. no. M0455), 1,3-dimethylurea (cat. no. D0289), and 3-phenylpropionic acid (cat. no. H0183) were purchased from Tokyo Chemical Industry (Tokyo, Japan). LysoTracker Green DND-26 (LysoTracker, cat. no. L5726), Quant-iT PicoGreen (PicoGreen, cat. no. P11496), and Quant-iT OliGreen (OliGreen, cat. no. O7582) were purchased from Thermo Fisher Scientific (Waltham, MA, USA). Ultrapure water (18.2 MΩ cm) obtained using a Millipore Direct-Q3 (Millipore, Billerica, MA, USA) system was used in all experiments. All chemicals were used as received without further purification unless otherwise stated.

1.2. Synthesis of Photoluminescent M^{n+}-doped DES (M^{n+}-DES). For the initial library screening, each HBA was evaluated by pairing it with B1 (urea) at a fixed 1:2 molar ratio. Upon identifying A7 (BTEAC) as the optimal HBA, it was subsequently combined with various HBDs at specific molar ratios: 1:2 for B1-B4 and 1:1 for B5-B10. Ultimately, B9 (BA) was selected as the optimal HBD to pair with A7 for subsequent syntheses. To synthesize a photoluminescent M^{n+}-DES, BTEAC (2.08 mmol, 0.4735 g) and BA (2.08 mmol, 0.2539 g) were mixed and heated at 115 ºC for 30 min until a colorless homogeneous, transparent liquid was obtained. Subsequently, an aqueous M^{n+} solution (10 µL, $[Pb^{2+}]$ = 100 mM ; for other M^{n+}s, $[M^{n+}]$ = 1 M) was added to the DES under vigorous mixing. To minimize residual water, the mixture was maintained at 115 °C before being allowed to cool to ambient temperature, affording the M^{n+}-DES. Alternatively, the M^{n+}-DES was synthesized by dissolving equal amounts of BTEAC and BA together with the corresponding metal salt in water (1 mL), followed by water removal by evaporation at temperatures above 100 ºC.

1.3. Synthesis of Benzyltriethylammonium Nitrate (BTEAN) Benzyltriethylammonium nitrate (BTEAN) was synthesized via an anion metathesis reaction between BTEAC and $AgNO_3$. BTEAC (10.0 g, 43.9 mmol) was dissolved in water (30 mL). Separately, a solution of $AgNO_3$ (7.46 g, 43.9 mmol) in water (20 mL) was prepared under light-shielded conditions to prevent photoreduction. The $AgNO_3$ solution was slowly added to the BTEAC solution with vigorous stirring, resulting in the immediate formation of a white precipitate of silver chloride (AgCl). The reaction mixture was stirred at 25 ºC for 30 min in the dark. The suspension was then filtered through a 0.45 µm cellulose acetate membrane filter to remove the AgCl precipitate. The absence of residual Ag^+ ions in the filtrate was subsequently verified by adding a few drops of an aqueous NaCl solution (2 M) to an aliquot of the clear solution, which yielded no observable precipitation or turbidity. The filtrate was concentrated using a rotary evaporator at 80 °C under reduced pressure to remove the solvent. The resulting viscous residue was further dried in a vacuum oven at 60 °C for 24 h to yield 8.69 g of BTEAN as a hygroscopic solid (77.8% yield). The obtained BTEAN was stored in a desiccator prior to use for DES preparation.

1.4. Synthesis of M^{n+}-DES Eutectogels. PVA eutectogels based on M^{n+}-DESs were prepared using a solvent-regulation method.[1] M^{n+}-DES (4.08 g) was mixed with water (4.08 mL) and PVA (0.72 g), corresponding to a 15 wt% PVA eutectogel. The mixture was vigorously stirred

at 85 ºC for 3 h. The mixture initially appeared opaque, but gradually transitioned into a colorless transparent solution, indicating the formation of a homogeneous gel precursor mixture. The mixture was then poured into a polytetrafluoroethylene (PTFE) mold and placed in an oven at 70 ºC for 18 h to remove water and form the PVA eutectogel. To enhance mechanical strength via the freeze-thaw method, the eutectogel was cooled to -20 ºC for 20 h and then heated at 100 ºC for 4 h. This freeze-thaw cycle was repeated twice. Finally, the resulting PVA eutectogels were cooled to 25 ºC for further applications. Poly(acrylic acid) (PAA), poly(2-hydroxyethyl acrylate) (PHEA), and poly(methacrylic acid) (PMAA) eutectogels were synthesized via thermal polymerization of their respective monomers in the presence of M^{n+}-DES. M^{n+}-DES (4.08 g) was combined with each monomer (0.72 g; corresponding to the 15 wt% polymer eutectogel) and stirred at 70 ºC for 10 min. The mixture was cooled to 25 ºC and supplemented with the crosslinker *N*,*N′*-methylenebisacrylamide (0.1 mol% relative to monomer) and the thermal initiator $K_2S_2O_8$ (2 mol% relative to monomer). The mixture was then poured into a PTFE mold and placed in an oven at 70 ºC for 18 h to complete polymerization. The resulting eutectogels were cooled to 25 ºC for further applications. Note that eutectogels with various polymer contents (e.g., 10, 20, or 30 wt%) can also be prepared by proportionally adjusting the initial amounts of polymer or monomer relative to the M^{n+}-DES, while maintaining the specified additive ratios.

1.5. Synthesis of Hydrogels. A 15 wt% PVA hydrogel was synthesized using the freeze-thaw cycling method. PVA (0.720 g) was dissolved in water (4.08 mL) and stirred at 85 ºC for 3 h. The resulting solution was poured into a PTFE mold and frozen at -20 ºC for 20 h, followed by thawing at 25 ºC for 4 h. This freeze-thaw cycle was repeated twice. A 15 wt% PMAA hydrogel was synthesized via thermal polymerization. Methacrylic acid (MAA; 0.72 g) was combined with water (4.08 mL) and stirred at 25 ºC for 10 min. The mixture was then supplemented with *N*,*N′*-methylenebisacrylamide (0.1 mol% relative to MAA) and $K_2S_2O_8$ (2 mol% relative to MAA). The final mixture was poured into a PTFE mold, tightly sealed to prevent dehydration, and heated in an oven at 70 ºC for 18 h.

1.6. Synthesis of M^{n+}-DES-in-Oil Emulsion. M^{n+}-DES (0.270 g) was combined with Triton X-100 (2.00 g) and vigorously stirred to achieve homogenization. Subsequently, 7.73 g of toluene was added to the mixture, which was vortexed for 1 min to obtain a M^{n+}-DES-in-oil emulsion.

1.7. Preparation of Multilevel Encryption Platforms Using PVA Eutectogels. A neat PVA 10 wt% eutectogel, acting as the encryption blank canvas, was prepared in a glass petri dish following the solvent-regulation method described above. Graphical information was encrypted onto the canvas by contact-transferring aqueous M^{n+} solutions ($[M^{n+}]$ = 1 M) using rubber stamps engraved with various motifs, including alphabet characters, squirrels, and floral patterns. The 5-level authentication platform was constructed by spatially arranging PVA eutectogels with specific M^{n+} combinations into 96-well microplates (225 mg per well), as detailed in the spatial map in Figure 8D. Optical decryption of the encrypted information was performed under ambient conditions via illumination with 254 nm and 365 nm UV lamps. Discrete emission channels were further isolated using commercial red and green optical bandpass filters to differentiate overlapping emission signals.

1.8. Instrumentation. Photoluminescence (PL) spectra of DESs and M^{n+}-DES eutectogels were collected using a Cary Eclipse fluorescence spectrophotometer (Agilent Technologies, Santa Clara, CA, USA). Ultraviolet-visible (UV-vis) absorbance and transmittance spectra of DESs were recorded using a Cary 100 UV-vis spectrophotometer (Agilent Technologies, Santa Clara, CA, USA). Raman spectra were collected using a LabRAM HR Evolution confocal Raman microscope (HORIBA, Kyoto, Japan). Absolute PL quantum yields (PLQYs) and time-resolved PL decay traces were measured using an FLS1000 PL spectrometer (Edinburgh Instruments, Livingston, UK, NFEC-2025-03-305119) under λ_{ex} = 320 nm for the Pb^{2+}-DES and λ_{ex} = 300 nm for the Eu^{3+}- and Tb^{3+}-DESs. The decay traces were monitored via time-correlated single-photon counting (TCSPC) for the Pb^{2+}-DES and multichannel scaling (MCS) for the Eu^{3+}- and Tb^{3+}-DESs. Attenuated total reflection Fourier-transform infrared (ATR-FT-IR) spectra were obtained using a Nicolet iS50 FT-IR spectrometer (Thermo Fisher Scientific, Waltham, MA, USA). X-ray diffraction (XRD) patterns were recorded using a X′Pert Pro diffractometer (Malvern Panalytical, Malvern, UK) using Cu Kα radiation (λ = 1.54178 Å). X-ray photoelectron spectroscopy (XPS) was carried out using a K-Alpha X-ray photoelectron spectrometer (Thermo Fisher Scientific, Waltham, MA, USA) equipped with a monochromated Al Kα X-ray source ($h\nu$ = 1486.6 eV). Temperature-dependent solid-state ^{13}C nuclear magnetic resonance (NMR) spectra were collected using a Bruker AVANCE NEO 400 MHz NMR spectrometer (Bruker, Billerica, MA, USA). Uniaxial tensile tests were performed using a MultiTest 2.5-dV universal testing machine (Mecmesin, Slinfold, UK) at a strain rate

of 100 mm min^{-1}. To investigate the self-healing properties of the PVA eutectogels, two discrete PVA 15 wt% eutectogel segments were brought into physical contact and incubated at 90 °C for 30 min. The mechanical integrity and tensile recovery of resulting self-healed eutectogel were subsequently quantified using a universal testing machine. The contact angle was measured at 25 °C using a Phoenix 150 contact angle goniometer (SEO, Suwon-si, Republic of Korea) based on the sessile drop method. Thermogravimetric analysis (TGA) was conducted using a NEXTA STA200RV (Hitachi High-Tech Science, Tokyo, Japan) in an N_2 atmosphere at a heating rate of 10 °C min^{-1}. Differential scanning calorimetry (DSC) experiments were recorded using a DSC 7020 calorimeter (Hitachi High-Tech Science, Tokyo, Japan) in an N_2 atmosphere at a heating rate of 20 °C min^{-1}. The synthesized emulsion was imaged using an LSM700 confocal laser scanning microscope (CLSM; Carl Zeiss, Jena, Germany). Hydrodynamic diameters of M^{n+}-DES droplets in the emulsion were measured using a Zetasizer Nano ZS90 (Malvern Panalytical, Malvern, UK).

1.9. Molecular Dynamics (MD) Simulations. Molecular dynamics (MD) simulations were performed using the GROMACS 2023.3 software package to investigate the spontaneous structural evolution of the Pb^{2+}-doped DES. The initial topology and force field parameters for benzoic acid (BA) and benzyltriethylammonium ($BTEA^+$) cations were generated via the LigParGen server, employing the OPLS-AA force field combined with the 1.14*CM1A charge model to accurately describe the intermolecular interactions.[2-4] The nonbonded parameters for the Cl^- ions were taken from the standard OPLS-AA force field library.[5] Furthermore, the nonbonded Lennard-Jones parameters and point charges for the Pb^{2+} ions were adopted from the standard OPLS-AA parameters established for divalent heavy metal cations to accurately capture the metal-ligand coordination interactions.[6] The simulation box was constructed to mimic the experimental liquid environment, containing 500 $BTEA^+$ cations, 500 BA molecules, 520 chloride (Cl^-) ions, and 10 Pb^{2+} ions. Initial configurations were generated by randomly inserting molecules into a cubic box. The system underwent energy minimization using the steepest descent algorithm until the maximum force converged to below 1000 kJ mol^{-1} nm^{-1}. Following minimization, equilibration was conducted in two phases: first in the NVT ensemble to stabilize the temperature, followed by the NPT ensemble to achieve the correct liquid density. Although the optical measurements were performed at room temperature, the simulation temperature was maintained at 353.15 K (80 °C) using the V-rescale thermostat. This elevated temperature condition was strategically applied to overcome the inherently high viscosity of

the DES, ensuring accelerated dynamics and sufficient phase-space sampling for the spontaneous formation of the coordination complexes. The pressure was kept at 1 bar using the stochastic cell rescaling (C-rescale) barostat, which was deliberately chosen over traditional methods to ensure accurate volume fluctuations without unphysical pressure oscillations.[7] Long-range electrostatic interactions were calculated using the Particle Mesh Ewald (PME) method with a cutoff of 1.0 nm, and van der Waals interactions were treated with the same 1.0 nm cutoff. All bond lengths involving hydrogen atoms were constrained using the LINCS algorithm. Finally, the production run was performed for 10 ns with a time step of 1 fs. Although 10 ns is relatively short for typical viscous DES systems, the elevated simulation temperature (353.15 K) significantly reduced the macroscopic viscosity and accelerated the local ionic diffusion. This ensured that the 10 ns timeframe was fully sufficient to achieve structural equilibration and sample the heavy metal coordination dynamics. The radial distribution functions (RDFs) and coordination numbers (CNs) were analyzed using the gmx rdf module to investigate the specific Pb-Cl interactions and the spontaneous formation of the 0D complexes.

1.10. Density Functional Theory (DFT) Calculations. To elucidate the PL mechanism and the heavy-atom effects of the structurally confined complexes, density functional theory (DFT) and time-dependent DFT (TD-DFT) calculations were performed using the ORCA program package (version 6.1.0). Instead of an idealized geometry, the initial coordinates of the 0D $[PbCl_4]^{2-}$ complex were directly extracted from the final snapshot of the 10 ns MD trajectory to accurately reflect the realistically distorted conformation induced by the DES hydrogen-bonding network. All ground-state and excited-state calculations were carried out using the PBE0 exchange-correlation functional coupled with Grimme's D3BJ dispersion correction. The def2-TZVP basis set was employed for the Cl atoms, while the SARC-ZORA-TZVP basis set was specifically assigned to the Pb atom. To rigorously account for the strong relativistic effects of the heavy lead atom, the Zero-Order Regular Approximation (ZORA) was incorporated. The highly polar nature of the DES matrix was implicitly described using the Conductor-like Polarizable Continuum Model (CPCM) parameterized for ethanol. The AutoAux procedure was utilized to automatically generate the appropriate auxiliary basis sets to accelerate the calculations. The absorption characteristics were evaluated via TD-DFT calculations on the S_0 geometry. Crucially, the spin-orbit coupling (SOC) effect was explicitly included (dosoc true) to identify the partially allowed transitions responsible for the prominent

310 nm excitation peak. To reveal the emission mechanism, the geometry of the lowest-lying triplet state (T_1) was fully optimized under the unrestricted Kohn-Sham (UKS) formalism to model the triplet self-trapped exciton (3STE) state driven by structural relaxation. Finally, the theoretical emission wavelength was computed by performing SOC-TD-DFT calculations on the fully optimized T_1 geometry (nroots = 15, including 15 singlet and 15 triplet states) to obtain the spin-forbidden $T_1 \rightarrow S_0$ phosphorescence transitions.

2. Supporting Figures

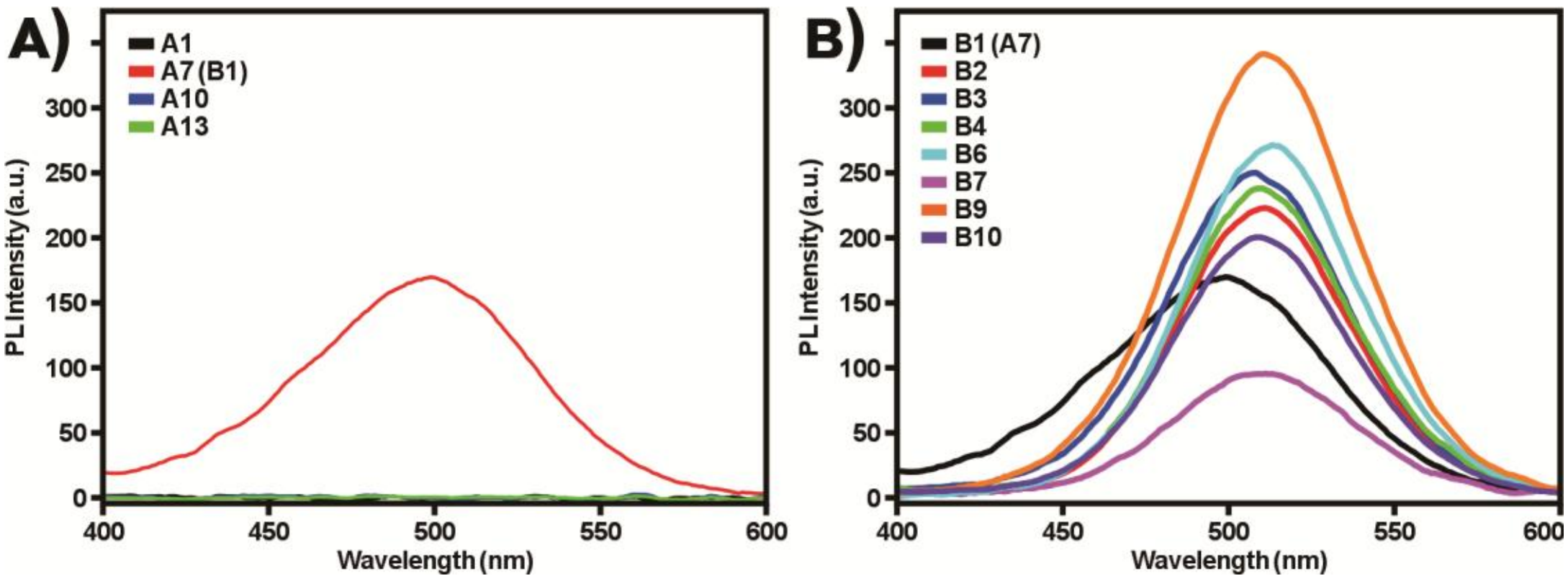

Figure S1. (A and B) Photoluminescence (PL) spectra of the Pb^{2+}-doped DES candidates consisting of (A) urea (B1) with various HBAs and (B) BTEAC (A7) with various HBDs (λ_{ex} = 320 nm). The spectra correspond to the homogeneous eutectic combinations highlighted in Figures 1C and 1D in the main text, respectively.

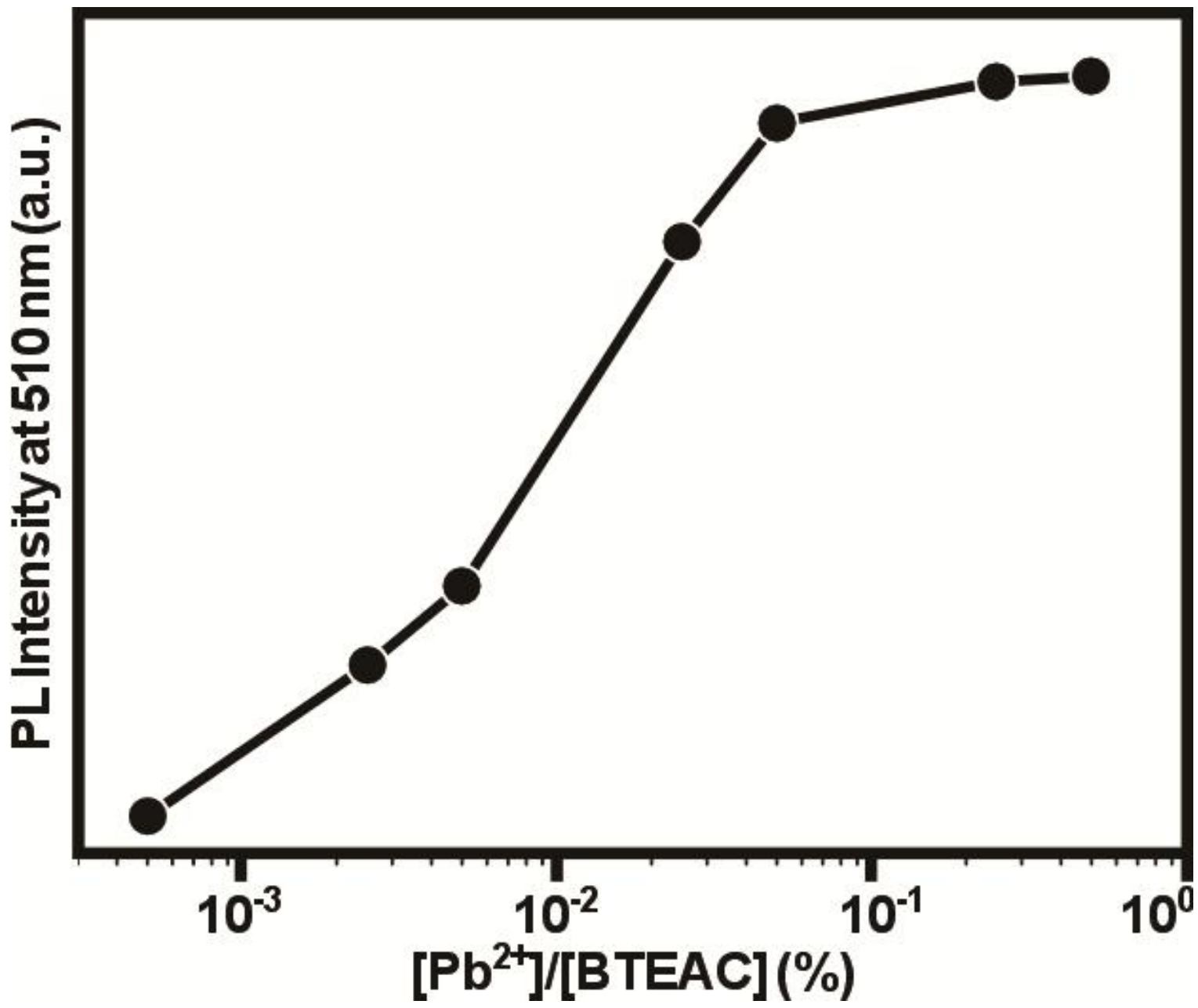

Figure S2. Dependence of PL intensity of the Pb^{2+}-DES on the molar fraction of Pb^{2+} relative to the BTEAC within the DES matrix. Measurements were recorded at the peak emission wavelength (510 nm).

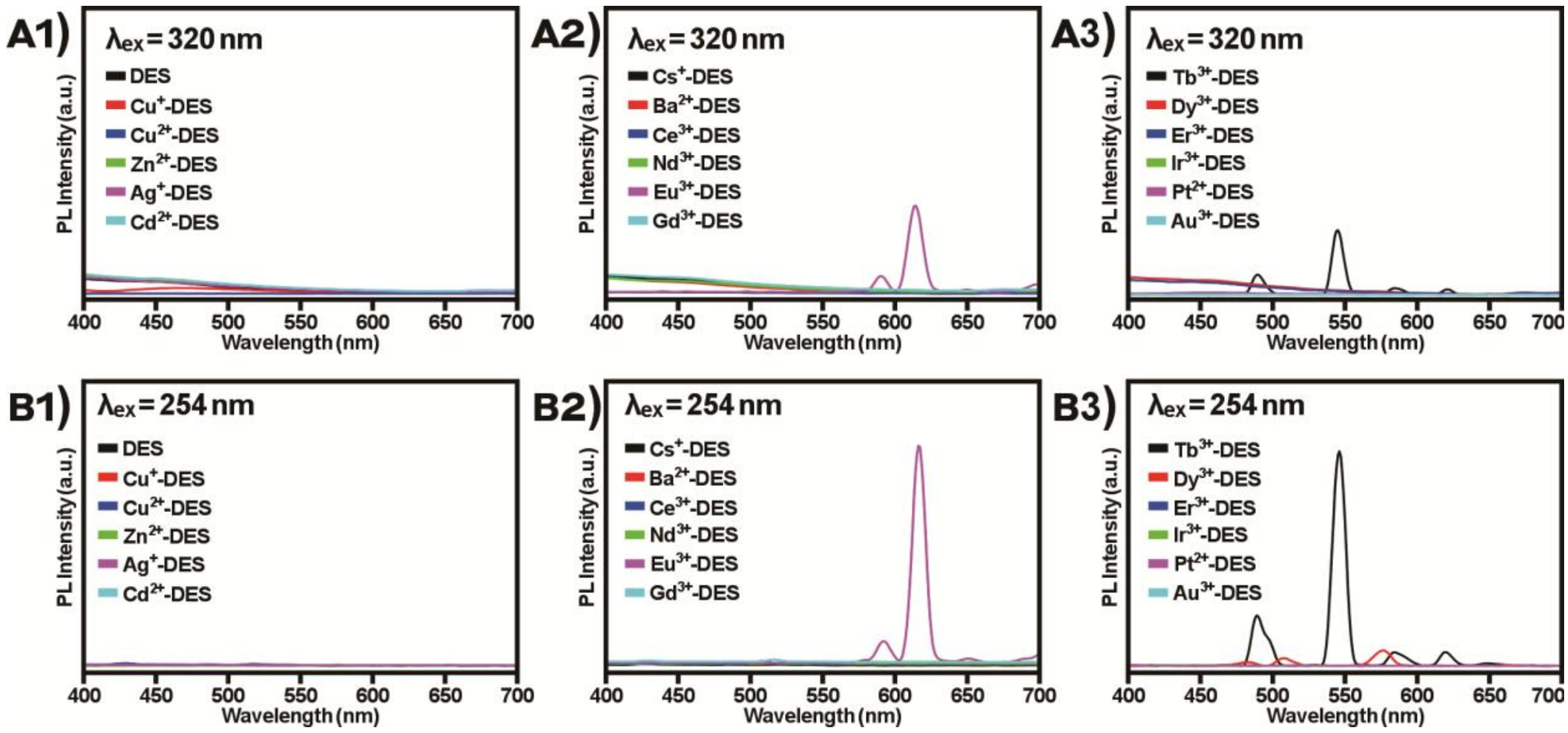

Figure S3. Representative PL spectra of the neat DES and a comprehensive series of M^{n+}-DESs ($M^{n+} = Cu^{+}$, Cu^{2+}, Zn^{2+}, Ag^{+}, Cd^{2+}, Cs^{+}, Ba^{2+}, Ce^{3+}, Nd^{3+}, Eu^{3+}, Gd^{3+}, Tb^{3+}, Dy^{3+}, Er^{3+}, Ir^{3+}, Pt^{2+}, and Au^{3+}) recorded under (A1 to A3) λ_{ex} = 320 nm and (B1 to B3) λ_{ex} = 254 nm. This systematic screening was performed to evaluate the cation-specific emission profiles and identify candidates for wavelength-dependent photoluminescent platforms.

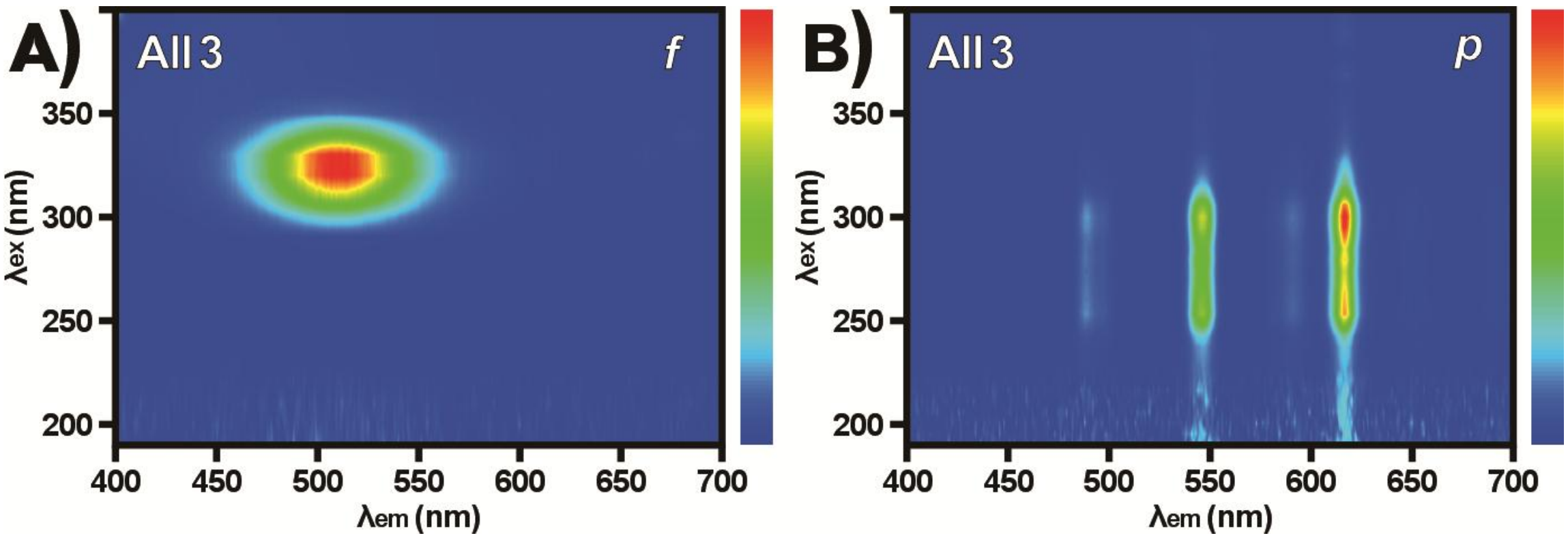

Figure S4. (A and B) Normalized EEM contour plots of the "All 3" system recorded in (A) fluorescence mode (*"f"*) and (B) phosphorescence mode (*"p"*).

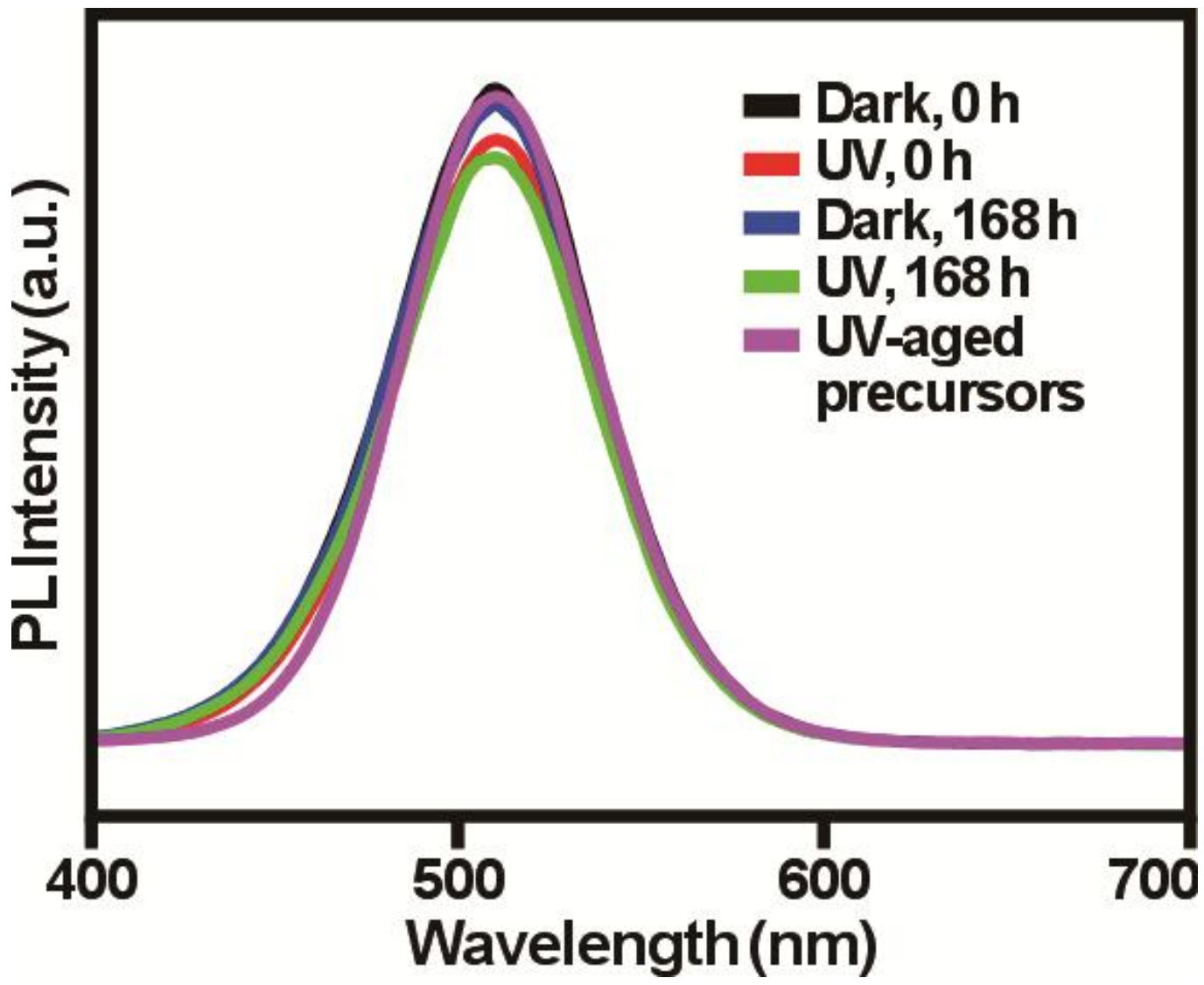

Figure S5. PL spectra of Pb^{2+}-DESs before and after 168 h of incubation under ambient dark conditions and continuous 365 nm UV irradiation. The minimal deviation in spectral profile and intensity after prolonged UV exposure demonstrates the high chemical integrity and long-term photostability of the Pb^{2+}-DES.

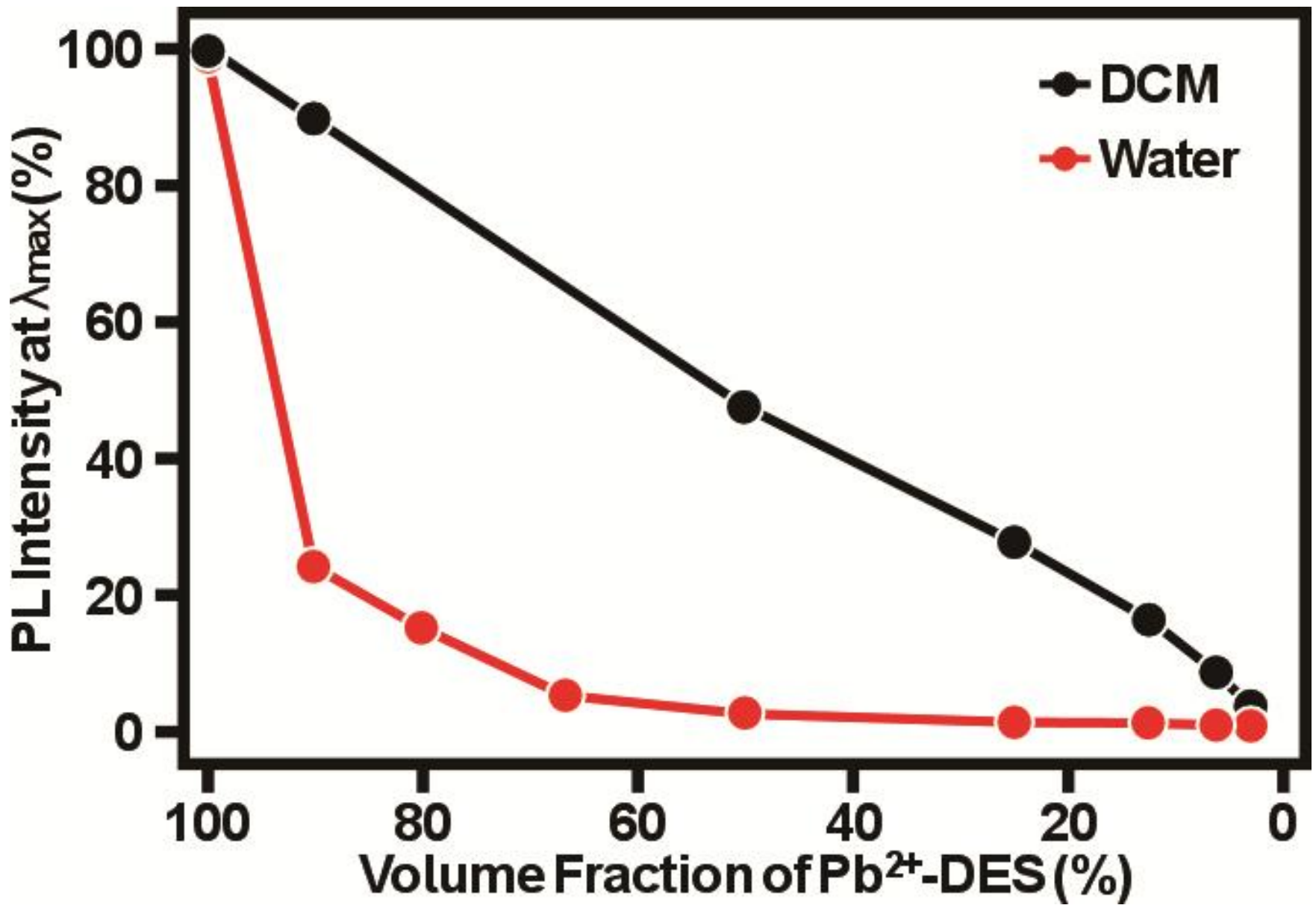

Figure S6. Relative PL intensity of the Pb^{2+}-DES upon dilution with dichloromethane (DCM) and water as a function of the Pb^{2+}-DES volume fraction (λ_{ex} = 320 nm, λ_{max} = 510 nm). The divergent trends, linear attenuation in DCM versus non-linear quenching in water, elucidate the critical role of the hydrogen-bonding network in maintaining the emissive state of the Pb^{2+} center.

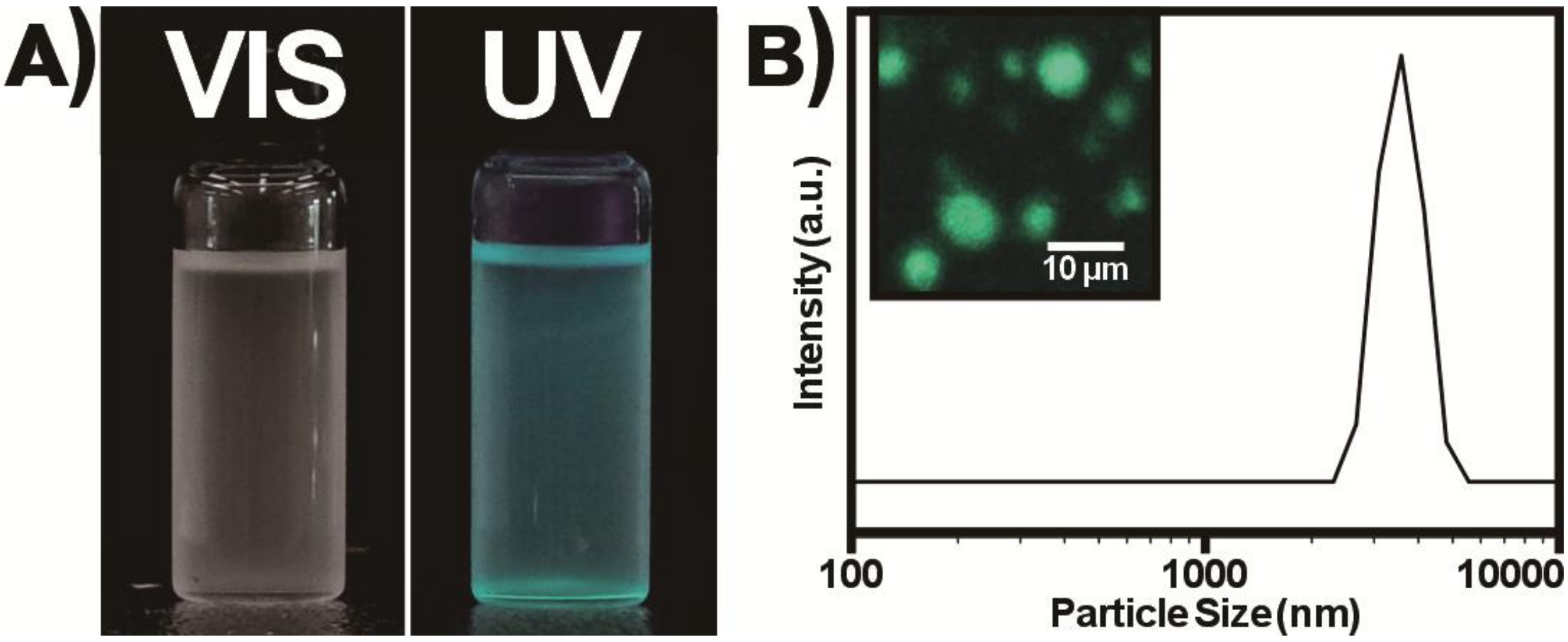

Figure S7. (A) Photographs of a Pb^{2+}-DES-in-oil (toluene) emulsion under ambient visible light (VIS) and 365 nm UV irradiation. (B) Particle size distribution of the Pb^{2+}-DES-in-oil emulsion characterized by dynamic light scattering (DLS). The inset displays confocal fluorescence images confirming the presence of spherical Pb^{2+}-DES droplets within the continuous toluene phase.

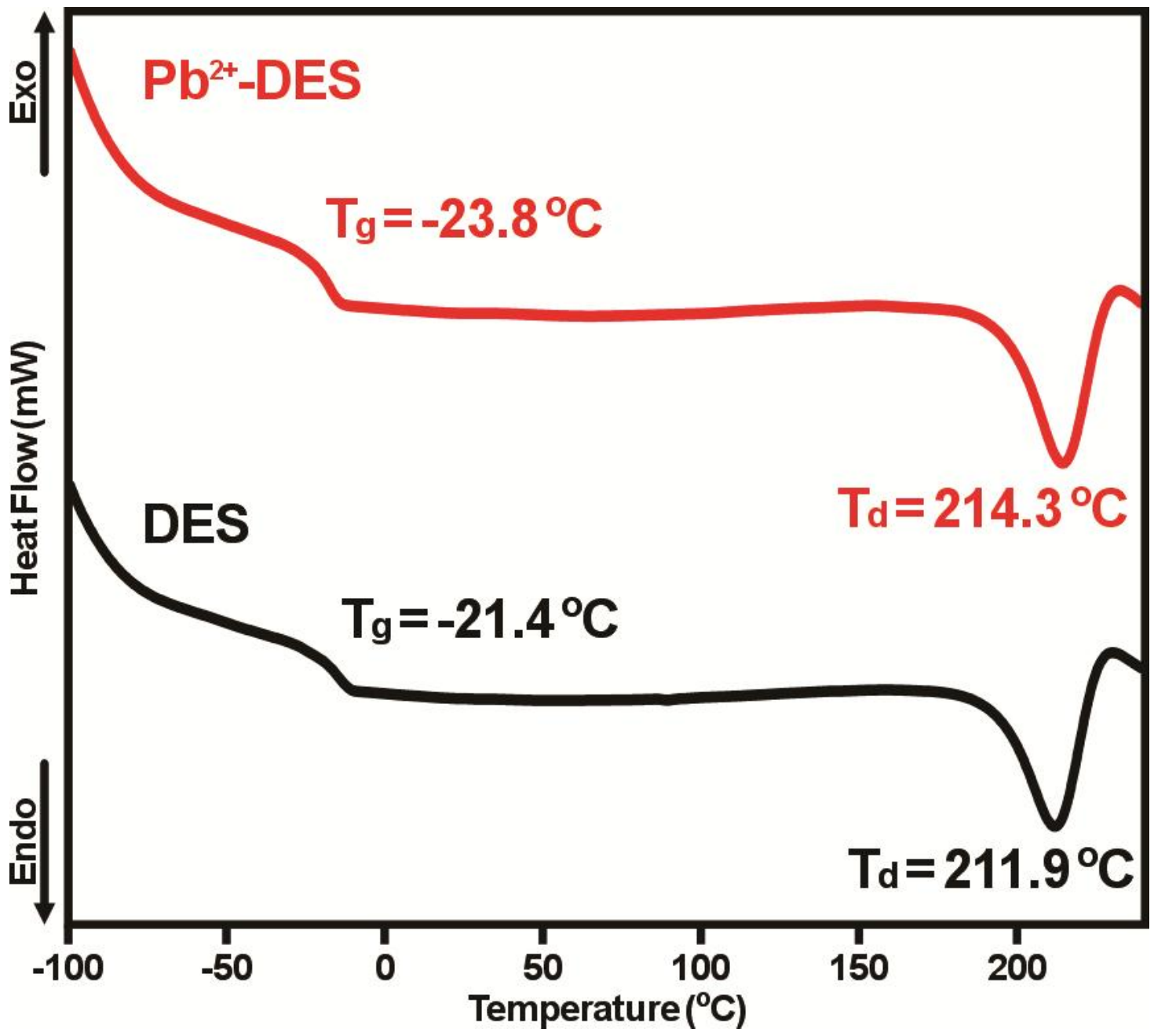

Figure S8. DSC thermograms of the neat DES and Pb^{2+}-DES. T_g and T_d refer to glass transition temperature and thermal decomposition temperature, respectively. Both the neat and Pb^{2+}-doped DESs exhibited a glass transition from amorphous solid phase to liquid phase at -21.4 ºC and -23.8 ºC, respectively, without crystalline melting transitions. The endothermic peaks observed at 211.9 ºC and 214.3 ºC were attributed to the thermal decomposition of the DES matrices, as supported by the significant mass loss observed in the TGA profiles in Figure 7F.

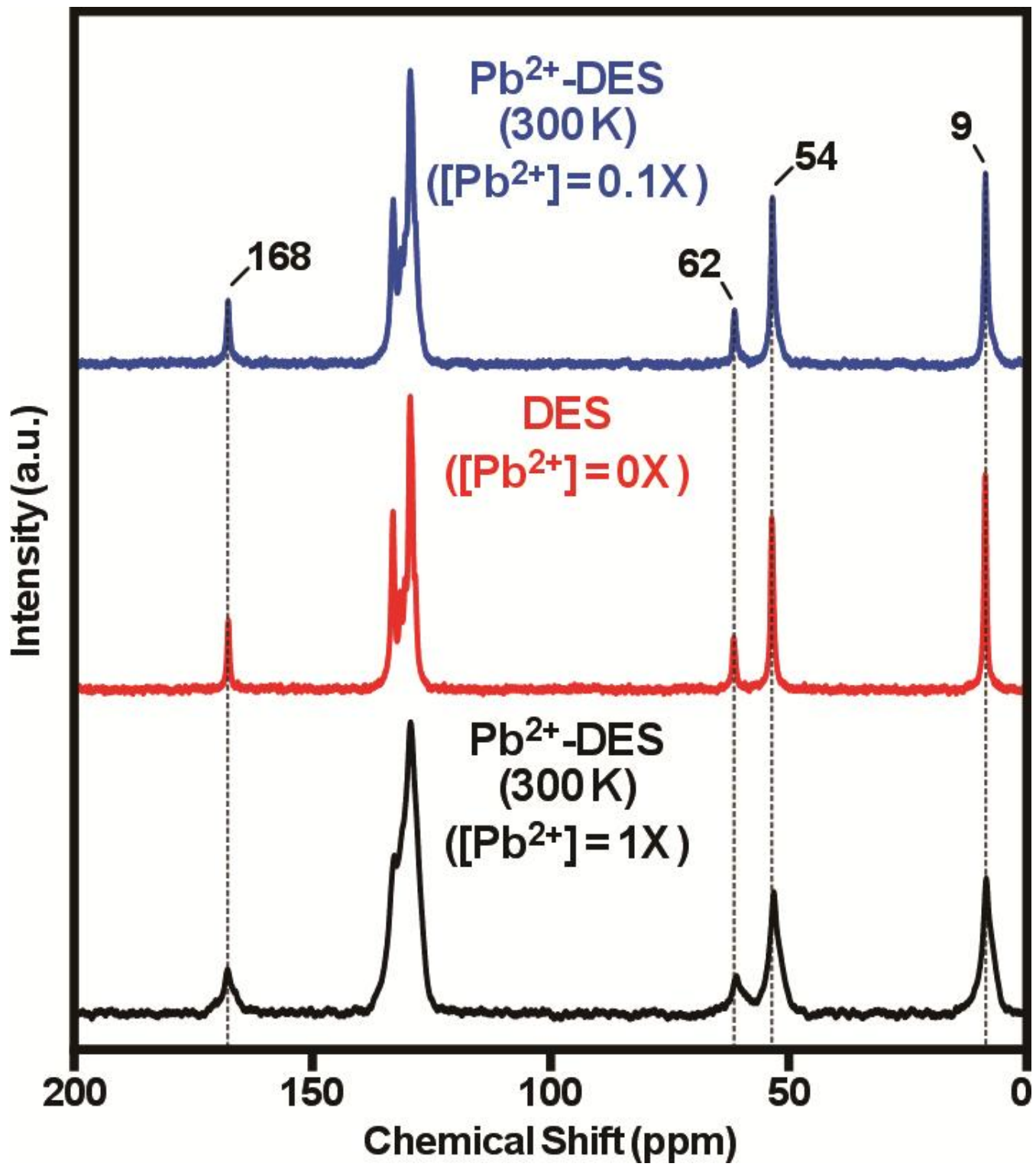

Figure S9. Solid-state ^{13}C NMR spectra of the Pb^{2+}-DES as a function of Pb^{2+} concentration (0X, 0.1X, and 1X), obtained at 300 K. Note that the spectra of the 0X (neat) DES and the 1X Pb^{2+}-DES (300K) are reproduced here from Figure 4D. Note that 1X corresponds to 0.05 mol% relative to BTEAC and BA.

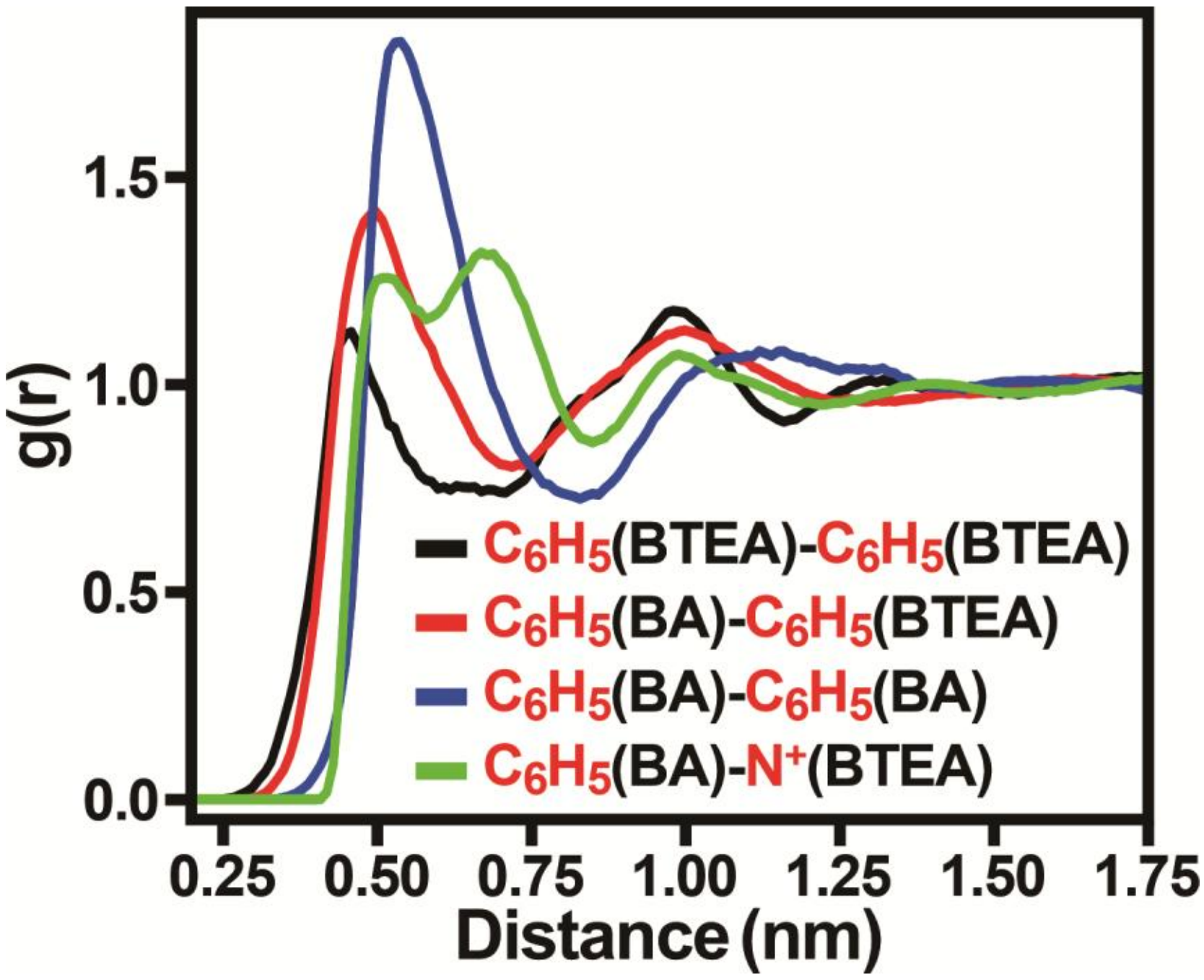

Figure S10. RDFs illustrating the specific interactions between the aromatic rings and the quaternary nitrogen center of the DES components.

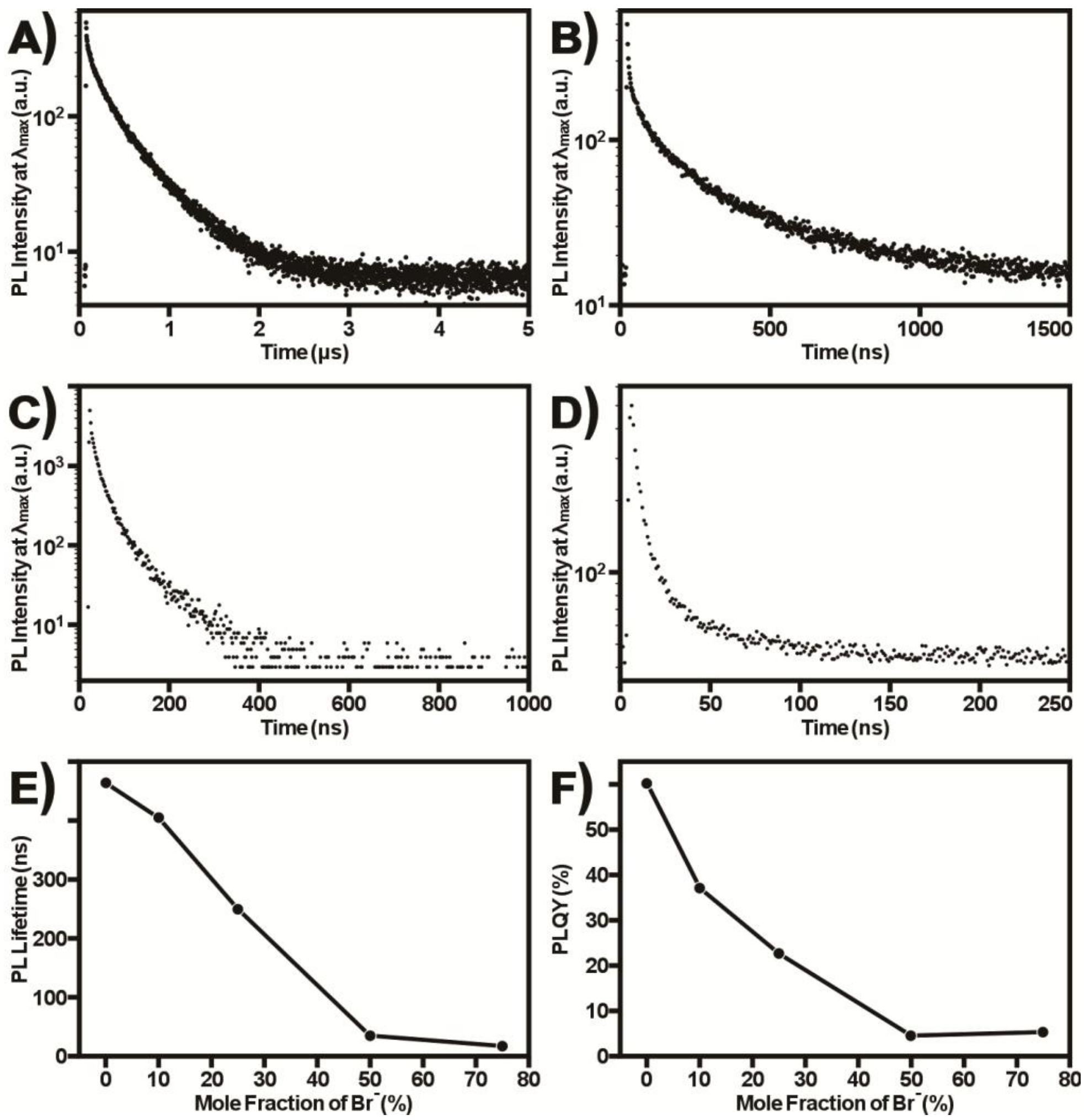

Figure S11. (A to D) TR-PL decay curves of mixed-halide Pb^{2+}-DESs, wherein (A) 10, (B) 25, (C) 50, and (D) 75 mol% of the chloride precursor (BTEAC) was substituted with the bromide analog (BTEAB) (λ_{ex} = 293 nm, λ_{em} = 510 nm). (E) Average PL lifetimes (λ_{ex} = 293 nm) and (F) PLQYs (λ_{ex} = 320 nm) of the Pb^{2+}-DESs as a function of Br^- mole fraction.

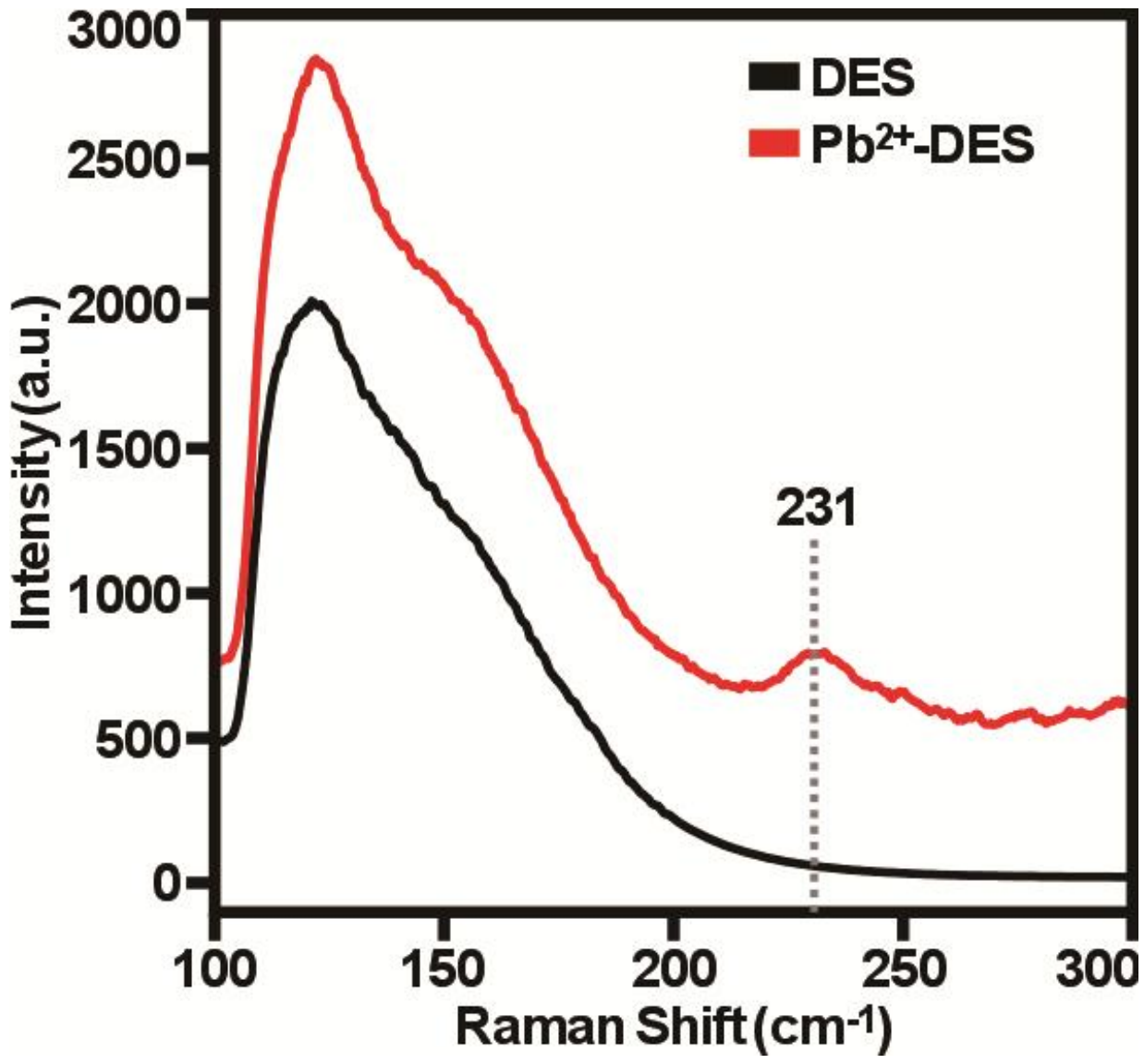

Figure S12. Raman spectra of the neat DES and Pb^{2+}-DES

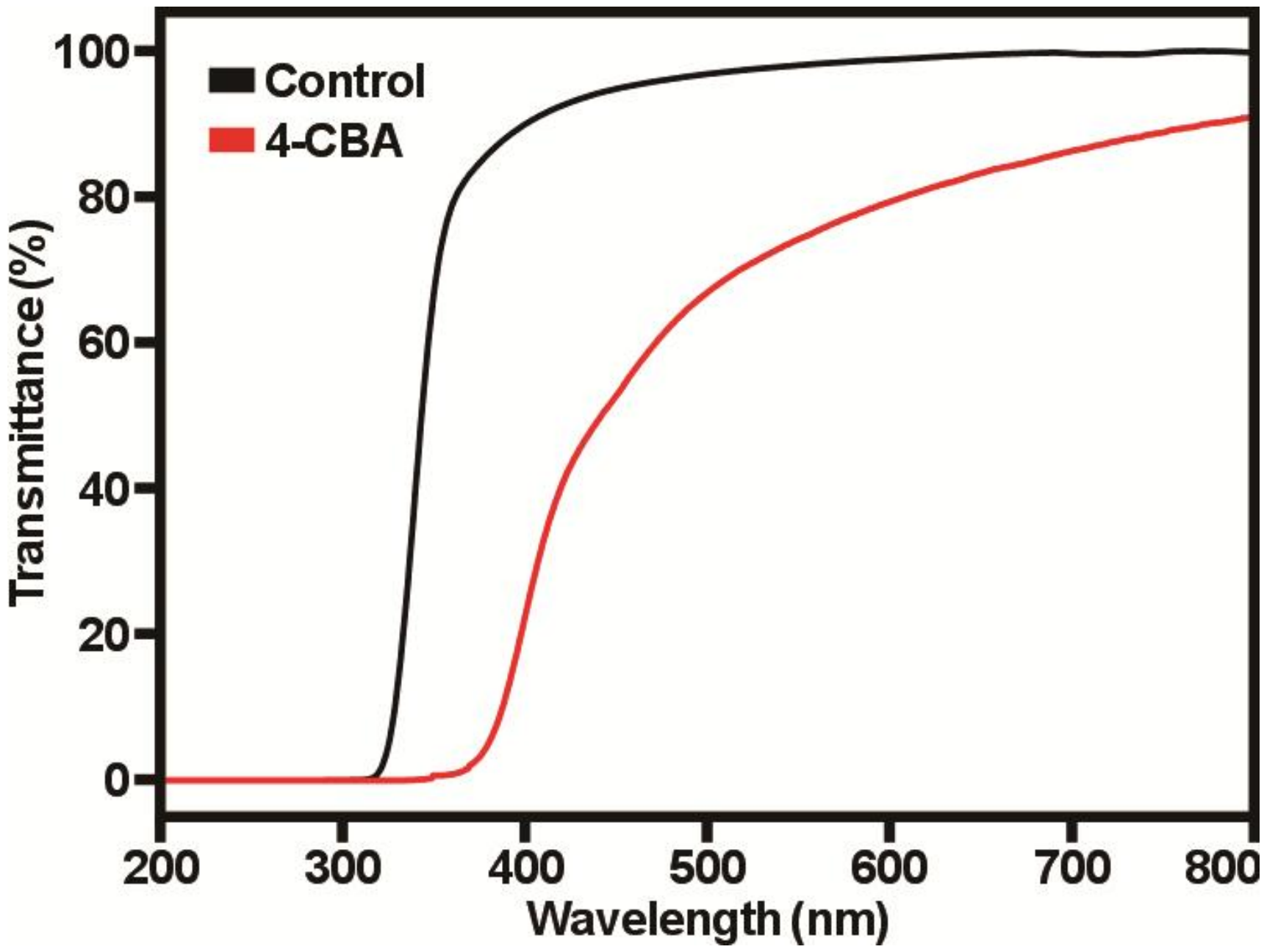

Figure S13. UV-vis transmittance spectra of Pb^{2+}-DESs formulated with BTEAC/BA (denoted as the control) and BTEAC/4-CBA.

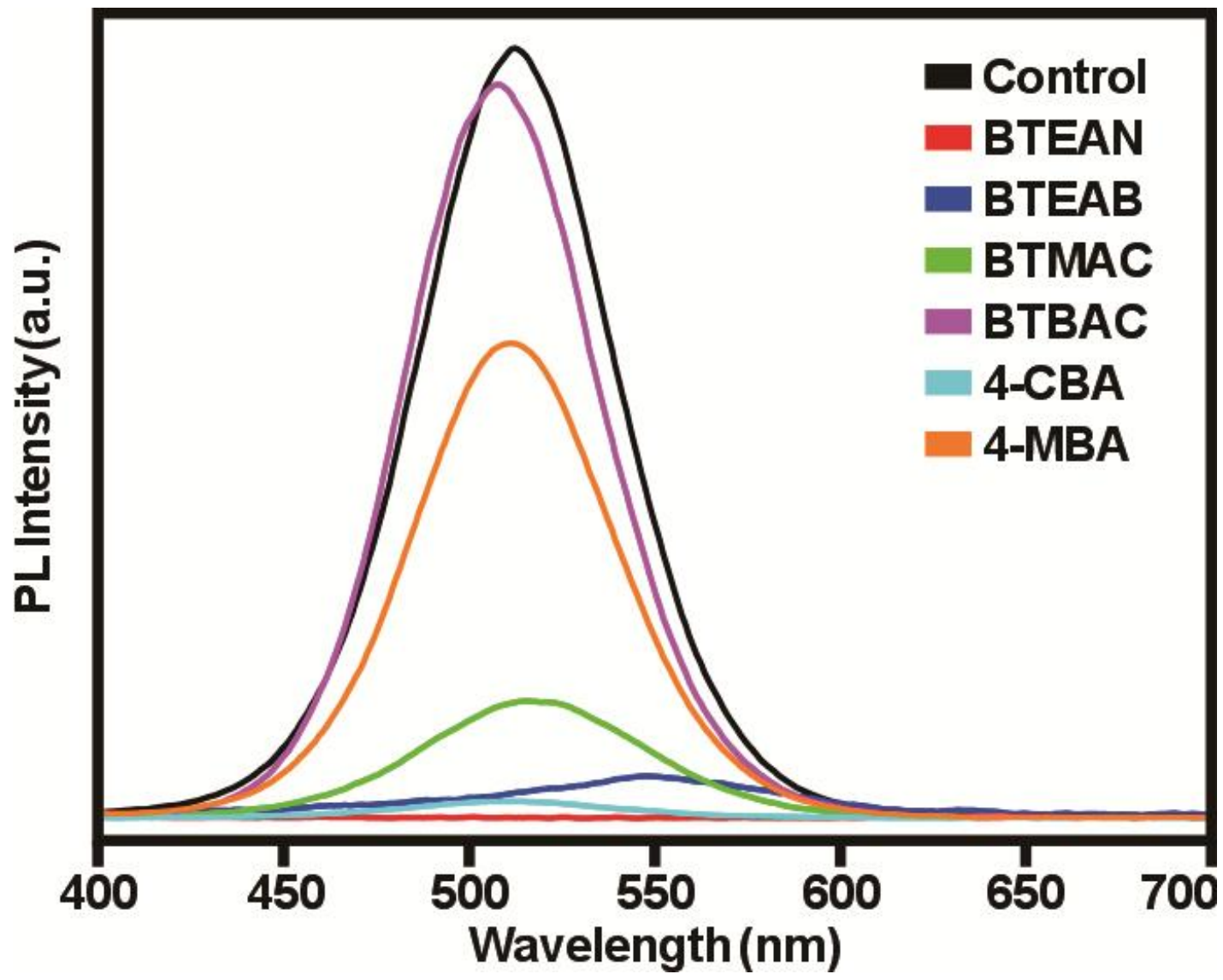

Figure S14. PL spectra of the Pb^{2+}-DES series used for the experimental validation of MD and DFT predictions, including the comprehensive dataset of BA with varying HBAs and BTEAC with varying HBDs evaluated in Figure 6 (λ_{ex} = 320 nm).

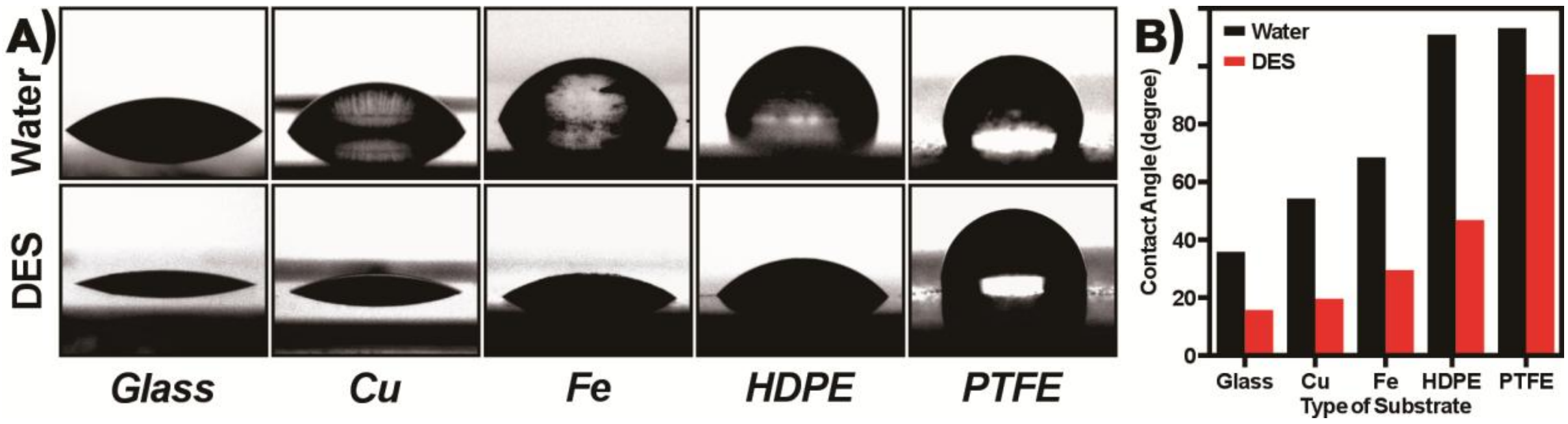

Figure S15. (A) Contact angle goniometry images of water and the neat DES sessile drops on a diverse series of hydrophilic (glass, Cu, and Fe) and hydrophobic (HDPE and PTFE) substrates. (B) Quantitative comparison of contact angles for water and DES across the investigated surfaces. The DES consistently exhibited lower contact angles relative to water across all substrates investigated, demonstrating superior wettability and amphiphilic behavior that facilitates robust interfacial contact on both polar and non-polar surfaces.

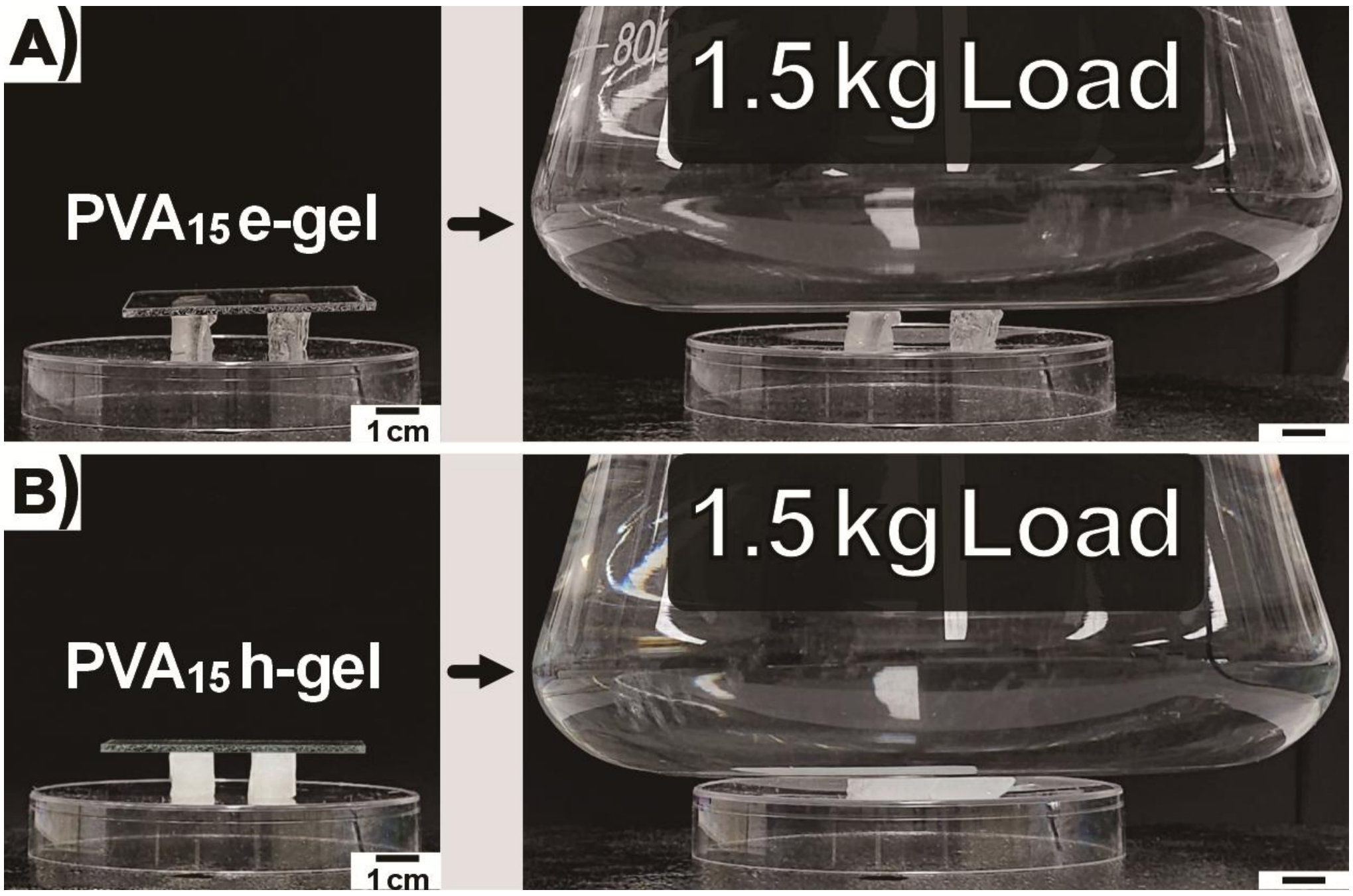

Figure S16. (A and B) Photographs of the (A) PVA_{15} eutectogel and (B) PVA_{15} hydrogel before and after the application of a 1.5 kg compressive load, demonstrating the superior mechanical integrity and load-bearing capacity of the eutectogel matrix.

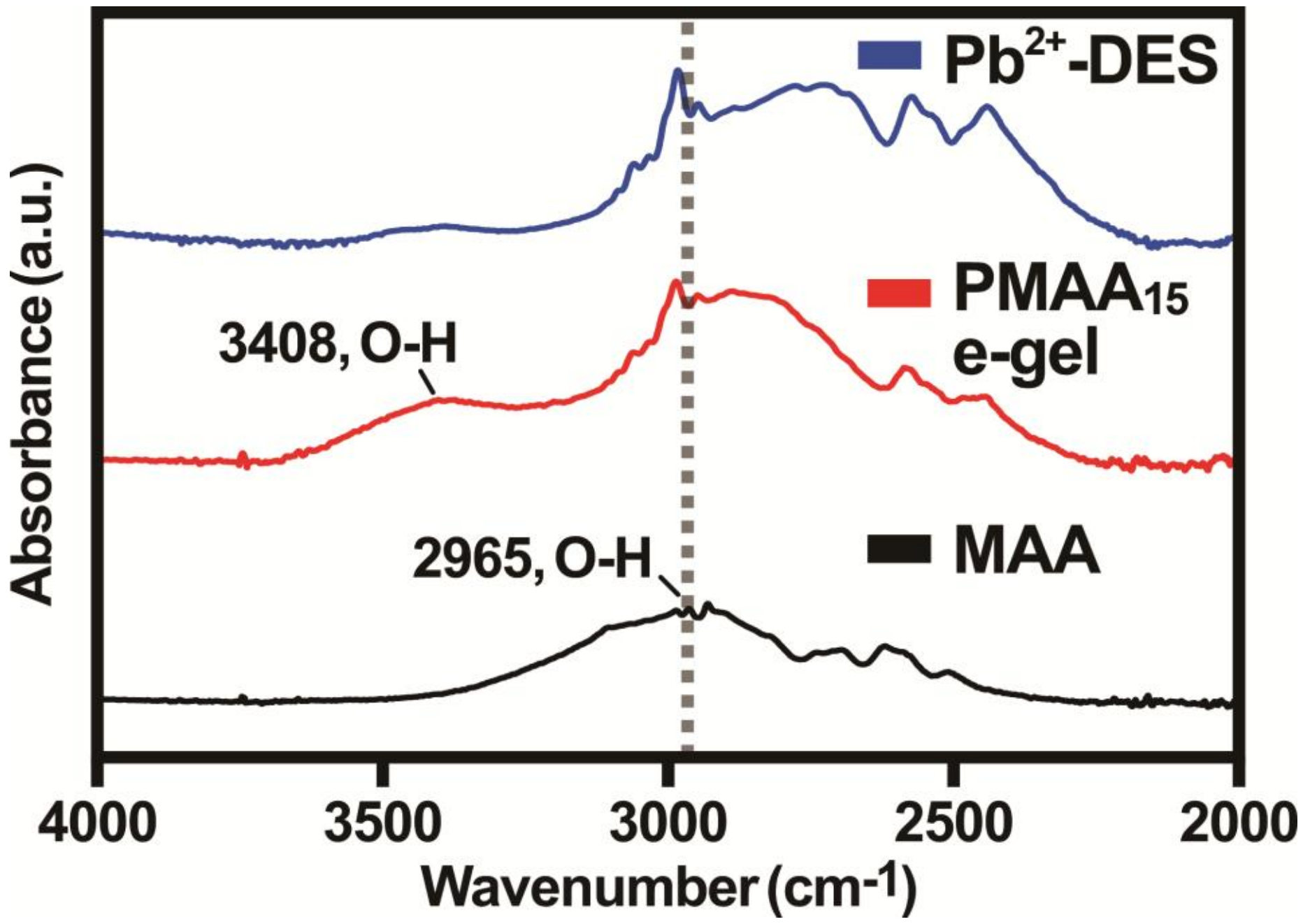

Figure S17. FT-IR spectra of the $PMAA_{15}$ eutectogel compared to their respective precursors (Pb^{2+}-DES and MAA) in the high-wavenumber region (4000 ~ 2000 cm^{-1})

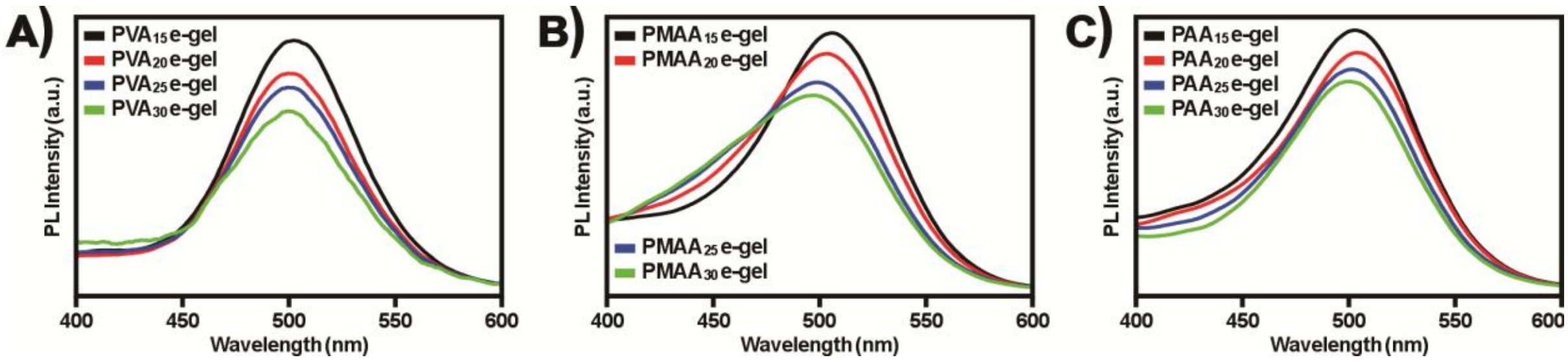

Figure S18. (A to C) PL spectra of the (A) PVA, (B) PMAA, and (C) PAA eutectogels as a function of polymer content (λ_{ex} = 320 nm).

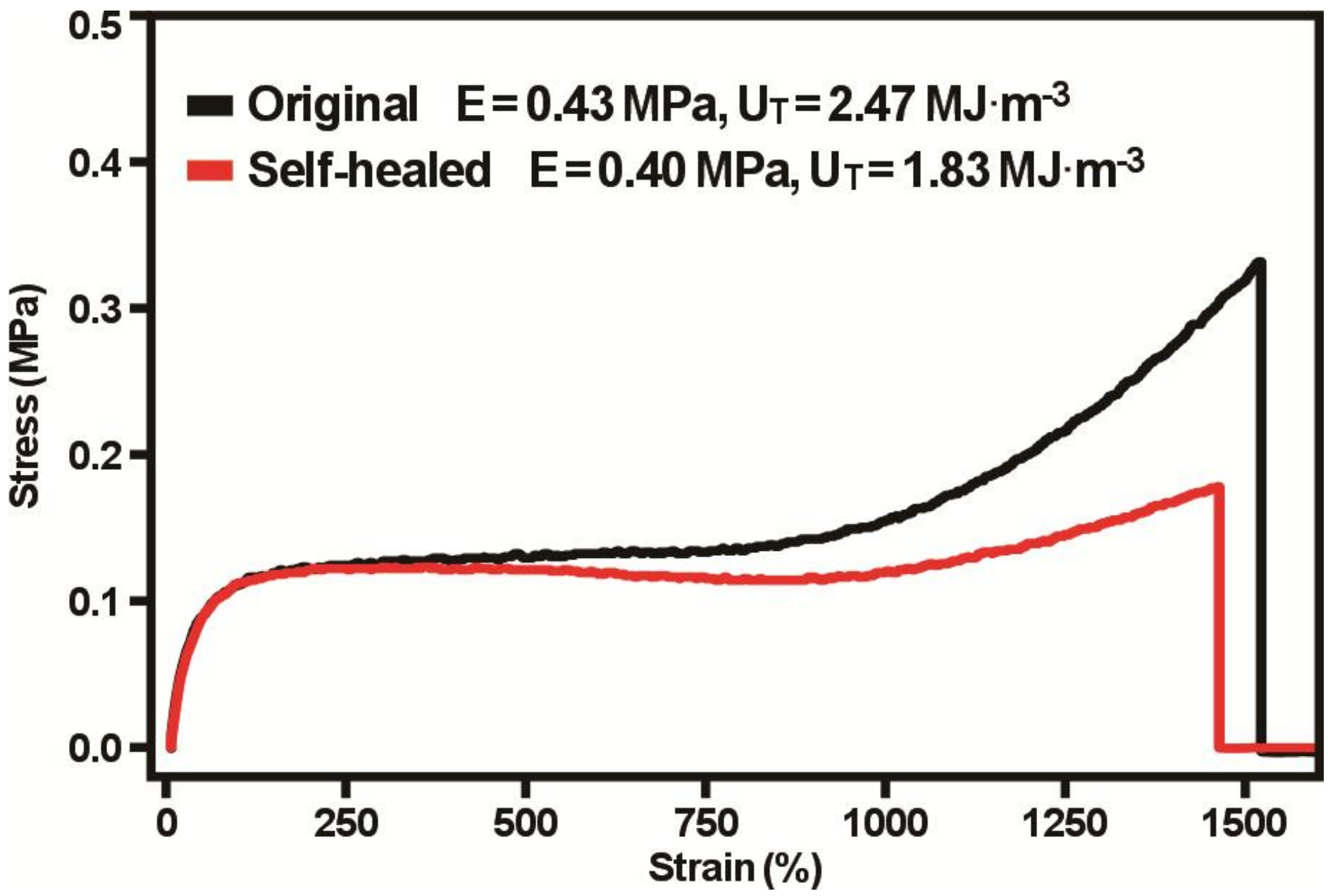

Figure S19. Tensile stress-strain curves of PVA_{15} eutectogel before and after the self-healing process. Following the healing event, the gel retained 93% of its original tensile modulus (E) and 74% of its initial toughness (U_T), confirming the high efficiency of the dynamic hydrogen bond network in restoring the structural integrity of the eutectogel matrix.

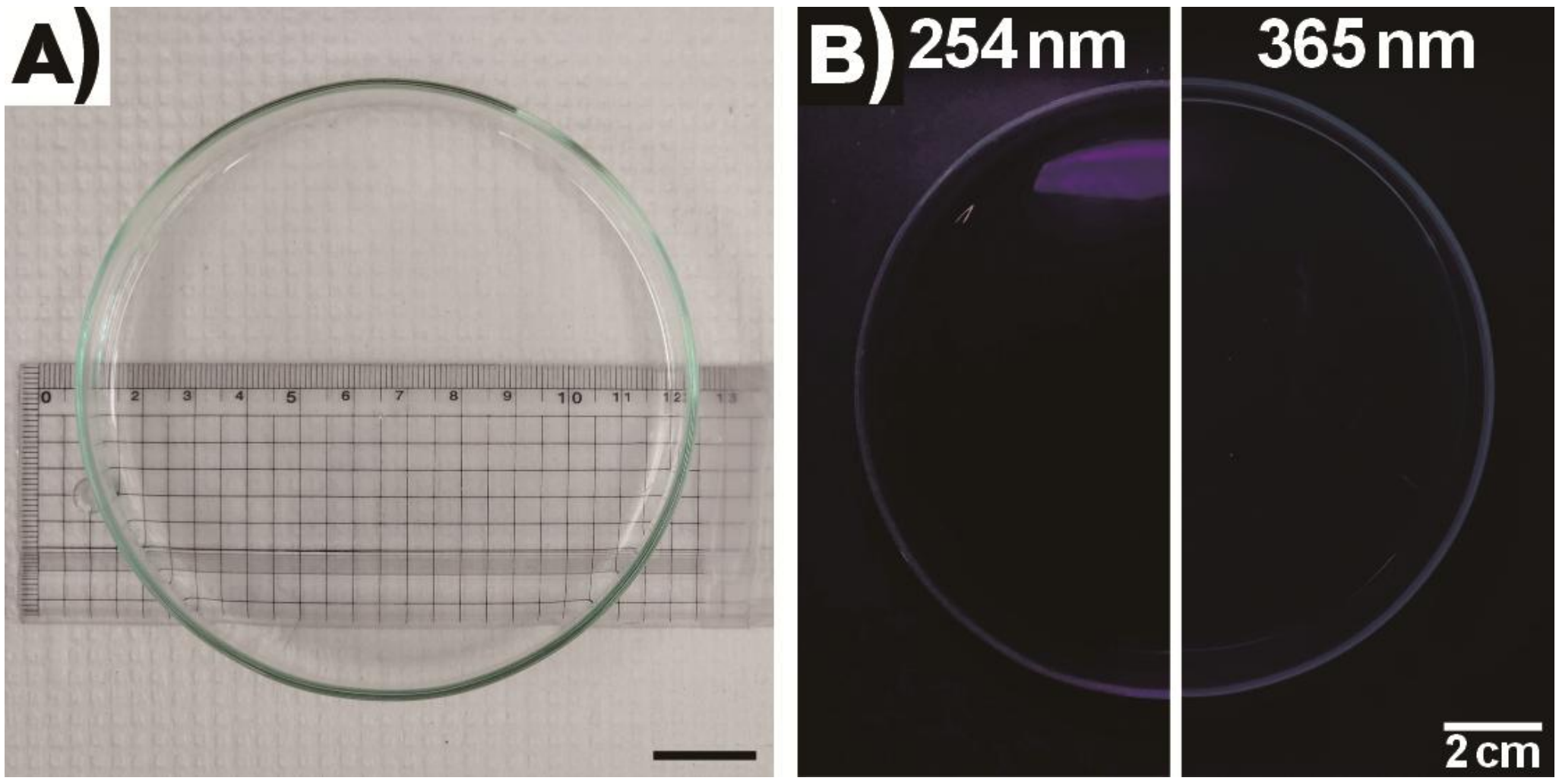

Figure S20. (A and B) Photographs of the neat PVA eutectogel "blank canvas" before M^{n+} stamping under (A) ambient visible light and (B) UV excitation (left: λ_{ex} = 254 nm, right: λ_{ex} = 365 nm), demonstrating its high optical transparency and featureless/colorless appearance prior to M^{n+} encryption.

3. Supporting Tables

Table S1. Direct comparison of the M^{n+}-DES platform with conventional emitters.

**Property**	**This work (M^{n+}-DES, BTEAC/BA)**	**Lead-halide perovskite nanocrystals (NCs) ($CsPbX_3$, X = Cl, Br, I)[8-11]**	**Cd-chalcogenide QDs (CdSe/ZnS)[12-14]**	**Cd-free QDs (InP/ZnS)[15-16]**	**Organic dyes (fluorescein or R6G)[17-18]**
**Active emissive species**	0D halometallate $[PbCl_4]^{2-}$ or Ln-Cl complex, in situ formed	3D semiconductor NCs	core/shell colloidal NCs	core/shell colloidal NCs	molecular π -systems
**Synthesis route**	single thermal mixing of two bulk salts + dopant; ambient air; no inert atmosphere	hot-injection or LARP; inert atmosphere; 150 to 230 °C	hot-injection in high-bp solvents; inert atmosphere; 200 to 300 °C	hot-injection; pyrophoric P precursors; inert atmosphere	multi-step organic synthesis + chromatography
**Raw material cost**	low (ammonium chloride salt + benzoic acid + ppm-level metal salt)	moderate (Cs, Pb, halide precursors + long-chain ligands)	moderate to high (Cd, Se precursors + ligands)	high (P precursors + ligands)	low to moderate per gram; high for high-grade laser dyes
**Equipment / energy footprint**	hotplate + magnetic stir; no purification needed	Schlenk line / glovebox; centrifugation; ligand exchange	Schlenk line / glovebox; multi-step purification	Schlenk line / glovebox; multi-step purification	rotary evaporator + column chromatography
**Heavy-metal content (active form)**	Pb^{2+} at ≈279.1 ppm; sequestered in $[PbCl_4]^{2-}$; Eu^{3+}/Tb^{3+} available as Pb-free alternatives	Pb in stoichiometric amounts within NC lattice (several tens of wt%)	Cd in stoichiometric amounts within NC core	no Cd or Pb; In is moderately toxic.	varies; many high-performing dyes contain halogenated/aromatic amines
**Solvent toxicity in synthesis**	none beyond the DES itself (low-volatility, biodegradable-class precursors)	toluene, hexane, octadecene, oleic acid, oleylamine	octadecene, oleic acid, trioctylphosphine oxide (TOPO)	octadecene, trioctylphosphine (TOP, pyrophoric)	DCM, chloroform, methanol; route-dependent
**PLQY (representative)**	60.2% (Pb^{2+}-DES); 2.9% (Eu^{3+}); 3.2% (Tb^{3+}); tunable	70 to 95% (best films and NCs)	50 to 90% (matured chemistry)	30 to 80%	10 to 95% depending on dye, solvent, and concentration
**Photostability (under continuous UV)**	no measurable PL loss after 168 h at 365 nm (Figure 3B)	minutes to hours without encapsulation; days to weeks when encapsulated	hours to days; photo-oxidation prone	hours; surface oxidation sensitive	minutes to hours (fluorescein loses ≈50% in 24 h, Figure 3B)
**Thermal stability**	operational to 90 °C with reversible response; decomposition at ≈214 °C	ion migration / phase segregation typically above 60 to 100 °C	ligand desorption above ≈80 °C; core degradation above ≈200 °C	ligand and surface degradation over ≈80 °C	thermal decomposition typically 150 to 300 °C; varies widely
**Air / moisture handling**	ambient; reversible hydration-dehydration cycling (Figure 3C)	moisture-sensitive; requires barrier coating	moderate moisture tolerance; oxidation under sustained UV in air	highly oxidation-sensitive surface	varies; many are water-stable but photo-bleach in air
**Color tunability**	metal-identity-tunable (Pb, Eu, Tb); excitation-wavelength-selective; combinable for additive mixing	halide-composition-tunable across visible; ion-exchange-prone	size-tunable; requires size-controlled synthesis	size-tunable; limited blue range	molecular-structure-tunable; requires new synthesis per color
**Processability into solids**	direct integration into eutectogels by gelation; printable, adhesive, self-healing	requires polymer/oxide encapsulation	polymer composites possible; ligand compatibility constraints	polymer composites possible	polymer matrices possible; concentration quenching common
**Multiplexing / time-gating**	intrinsic excitation-wavelength multiplexing + ns-to-ms lifetime contrast for time-gating	wavelength multiplexing; ns lifetimes	wavelength multiplexing; ns lifetimes	wavelength multiplexing; ns lifetimes	wavelength multiplexing; mostly ns lifetimes

Table S2. Vibrational mode assignments for the neat DES and its constituent precursors (BA and BTEAC).

Wavenumber (cm^{-1})			Vibrational Mode Assignment
DES	BA	BTEAC	
3059	3070	-	Stretching, carboxylic O-H + aromatic C-H
2983	-	2973	Stretching, aliphatic C-H
2574	2669	-	Stretching, carboxylic O-H + aromatic C-H
2437	2555	-	Stretching, carboxylic O-H + aromatic C-H
1698	1679	-	Stretching, carboxylic C=O
1381	1417	-	In-plane bending, carboxylic O-H
1225	1288	-	Stretching, aromatic ester C-O
1155	-	1153	Stretching, amine C-N
1009	-	1008	Stretching, amine C-N
755	-	769	Out-of-plane bending, aromatic C-H
706	704	714	Out-of-plane bending, aromatic C-H

Table S3. ^{13}C NMR peak assignments for BA, BTEAC, DES, and Pb^{2+}-doped DES samples. Note that the aromatic carbon signals originating from BA and BTEAC consistently appear in the region of approximately 130 ppm across all analyzed samples.

Sample	Chemical Shift (δ, ppm)	Peak Assignment
BA	172	Carboxyl carbon (C=O)
	125-134	Aromatic carbons (Ar-C)
BTEAC	128-136	Aromatic carbons (Ar-C)
	45-69	Benzylic carbon ($Ph-CH_2-N^+$)<br>Ethyl α-methylene carbons ($-CH_2-N^+$)
	6-16	Terminal methyl carbons ($Et-CH_3$)
DES	168	BA carboxyl carbon
	125-137	Aromatic carbons (Ar-C)
	62	Benzylic carbon ($Ph-CH_2-N^+$)
	54	Ethyl α-methylene carbons ($-CH_2-N^+$)
	9	Terminal methyl carbons ($Et-CH_3$)
Pb^{2+}-DES (300 K)	Comparable to DES	Corresponds to the respective DES carbons
Pb^{2+}-DES (400 K)	167	BA carboxyl carbon
	127-137	Aromatic carbons (Ar-C)
	68	Benzylic carbon ($Ph-CH_2-N^+$)
	47	Ethyl α-methylene ($-CH_2-$)
	9	Terminal methyl carbons ($Et-CH_3$)

Table S4. Optimized structural parameters (bond lengths and bond angles) of the highly distorted tetrahedral $[PbCl_4]^{2-}$ complex obtained from DFT calculations.

Parameter	Atoms	Value
Bond Length (Å)	Pb–Cl(1)	2.742
	Pb–Cl(2)	2.698
	Pb–Cl(3)	2.877
	Pb–Cl(4)	2.726
Bond Angle (º)	Cl(1)–Pb–Cl(2)	106.67
	Cl(1)–Pb–Cl(3)	105.03
	Cl(1)–Pb–Cl(4)	106.10
	Cl(2)–Pb–Cl(3)	138.26
	Cl(2)–Pb–Cl(4)	99.13
	Cl(3)–Pb–Cl(4)	97.42

Table S5. Selected calculated SOC-TD-DFT absorption transitions of the highly distorted 0D $[PbCl_4]^{2-}$ complex.

Excited State	Energy (eV)	Wavelength (nm)	Oscillator Strength (f_{osc})	Note
4	4.03	307.4	0.029	Experimental Excitation (310 nm)
7	4.88	253.8	0.057	Strong UV Absorption
8	4.94	250.6	0.041	Strong UV Absorption
11	5.31	233.5	0.066	Strong UV Absorption
28	5.70	217.4	0.123	Strong UV Absorption
32	5.74	215.8	0.042	Strong UV Absorption

Table S6. Calculated SOC-TD-DFT vertical emission transitions from the relaxed T_1 state to the S_0 ground state.

Excited State	Energy (eV)	Wavelength (nm)	Oscillator Strength (f_{osc})	Note
1	2.67	463.7	4.91×10^{-7}	$T_1 \rightarrow S_0$, Phosphorescence
2	2.67	463.7	4.91×10^{-7}	$T_1 \rightarrow S_0$, Phosphorescence
3	2.67	463.5	0.000	$T_1 \rightarrow S_0$, Phosphorescence

Table S7. Mechanical properties of the PVA, PMAA, and PAA eutectogels as a function of polymer content.

Gel	Tensile Modulus (MPa)	Toughness (MJ m^{-3})	Elongation at break (%)
PVA_{15} h-gel	0.04	0.11	184
PVA_{15} e-gel	0.41	2.38	1420
PVA_{20} e-gel	0.72	2.22	791
PVA_{25} e-gel	0.82	3.26	351
PVA_{30} e-gel	1.65	4.18	237
$PMAA_{15}$ e-gel	0.09	0.60	1480
$PMAA_{20}$ e-gel	0.90	2.96	910
$PMAA_{25}$ e-gel	2.48	2.80	500
$PMAA_{30}$ e-gel	5.47	3.83	406
PAA_{15} e-gel	0.03	0.45	3251
PAA_{20} e-gel	0.04	0.55	2765
PAA_{25} e-gel	0.09	0.94	1621
PAA_{30} e-gel	0.49	2.18	851

Table S8. Mechanical properties of the PVA eutectogels as a function of additional vacuum heat treatment duration.

Time (min)	Tensile Modulus (MPa)	Toughness (MJ m^{-3})	Elongation at break (%)
0	1.01	2.77	1442
30	0.98	3.89	1253
60	1.32	4.87	1153
120	1.28	5.19	1049
480	1.72	6.44	765

4. References

[1] S. Chen, J. Feng, *ACS Appl. Mater. Interfaces* **2023**, *15*, 44752–44762.

[2] W. L. Jorgensen, J. Tirado-Rives, *Proc. Natl. Acad. Sci. U. S. A.* **2005**, *102*, 6665–6670.

[3] L. S. Dodda, J. Z. Vilseck, J. Tirado-Rives, W. L. Jorgensen, *J. Phys. Chem. B* **2017**, *121*, 3864–3870.

[4] L. S. Dodda, I. Cabeza de Vaca, J. Tirado-Rives, W. L. Jorgensen, *Nucleic Acids Res.* **2017**, *45*, W331–W336.

[5] W. L. Jorgensen, D. S. Maxwell, J. Tirado-Rives, *J. Am. Chem. Soc.* **1996**, *118*, 11225–11236.

[6] A. S. de Araujo, M. T. Sonoda, O. E. Piro, E. E. Castellano, *J. Phys. Chem. B* **2007**, *111*, 2219–2224.

[7] M. Bernetti, G. Bussi, *J. Chem. Phys.* **2020**, *153*, 114107.

[8] L. Protesescu, S. Yakunin, M. I. Bodnarchuk, F. Krieg, R. Caputo, C. H. Hendon, R. X. Yang, A. Walsh, M. V. Kovalenko, *Nano Lett.* **2015**, *15*, 3692–3696.

[9] Q. A. Akkerman, G. Rainò, M. V. Kovalenko, L. Manna, *Nat. Mater.* **2018**, *17*, 394–405.

[10] A. Babayigit, A. Ethirajan, M. Muller, B. Conings, *Nat. Mater.* **2016**, *15*, 247–251.

[11] A. Loiudice, S. Saris, E. Oveisi, D. T. L. Alexander, R. Buonsanti, *Angew. Chem.-Int. Edit.* **2017**, *56*, 10696–10701.

[12] C. B. Murray, D. J. Norris, M. G. Bawendi, *J. Am. Chem. Soc.* **1993**, *115*, 8706–8715.

[13] R. Hardman, *Environ. Health Perspect.* **2006**, *114*, 165–172.

[14] A. M. Derfus, W. C. W. Chan, S. N. Bhatia, *Nano Lett.* **2004**, *4*, 11–18.

[15] P. Reiss, M. Carrière, C. Lincheneau, L. Vaure, S. Tamang, *Chem. Rev.* **2016**, *116*, 10731–10819.

[16] V. Brunetti, H. Chibli, R. Fiammengo, A. Galeone, M. A. Malvindi, G. Vecchio, R. Cingolani, J. L. Nadeau, P. P. Pompa, *Nanoscale* **2013**, *5*, 307–317.

[17] J. R. Lakowicz, *Principles of Fluorescence Spectroscopy*, 3 ed., Springer New York, NY, **2006**.

[18] C. Eggeling, J. Widengren, R. Rigler, C. A. M. Seidel, *Anal. Chem.* **1998**, *70*, 2651–2659.
